# Novel Combination Scalp Therapy for Androgenetic Alopecia: A Preliminary Retrospective Case Series with an Illustrative Four-Year Case

**DOI:** 10.3390/jcm15135055

**Published:** 2026-06-29

**Authors:** Jong-Hee Lee, Hyung Min Hahn

**Affiliations:** 1 Oakwood Bom Clinic, 46, Teheran-ro 87-gil, Gangnam-gu, Seoul 06164, Republic of Korea; ps887@hanmail.net; 2 Department of Plastic and Reconstructive Surgery, Ajou University School of Medicine, 164 World Cup-Ro, Yeongtong-Gu, Suwon-Si 16499, Gyeonggi-Do, Republic of Korea

**Keywords:** androgenetic alopecia, trichoscopy, quantitative dermoscopy, platelet-rich plasma, stromal vascular fraction, botulinum toxin, hair regeneration

## Abstract

**Background/Objectives**: Androgenetic alopecia (AGA) responds only partially to pharmacologic monotherapy. Combination procedural regimens incorporating platelet-rich plasma (PRP), stromal vascular fraction (SVF), and botulinum toxin (BTX) have been reported, but objective quantitative trichoscopic data on multimodal single-session protocols are limited. We retrospectively quantified the trichoscopic response to a four-component single-session scalp procedure used in routine clinical practice. **Methods**: Fifty-one consecutive AGA patients underwent a single-session procedure combining partial temporalis muscle resection with silicone implantation, negative-pressure scalp stimulation, and BTX, PRP, and SVF injections; 28 completed ≥ 4-month follow-up. Standardized 60× videodermoscopy at five predefined scalp locations was archived for paired quantitative analysis in six patients (30 location pairs, of which 28 were analyzable after excluding two pairs for motion artifact), with one additional patient imaged at four years. Six trichoscopic outcomes were derived by automated image analysis (Otsu thresholding, skeletonization, distance-transform shaft thickness); the primary analysis was performed at the patient level (*n* = 6) and a supporting analysis at the panel level (*n* = 28), each using paired Student’s *t*-tests. **Results**: In the primary patient-level analysis (*n* = 6 patients), five of six trichoscopic outcomes improved significantly at 3–4-month follow-up, each with a large effect size: median shaft thickness +54% (*p* = 0.025), terminal-hair proportion +52% (*p* = 0.028), vellus-hair proportion −33% (*p* = 0.011), diameter heterogeneity −14% (*p* = 0.017), and mean shaft thickness +33% (*p* = 0.029); hair coverage increased but did not reach statistical significance (+11%, *p* = 0.125). The supporting panel-level analysis (*n* = 28 paired panels) was concordant in direction and significant for all six metrics. In a single illustrative case followed for four years (*n* = 1; exploratory), mean shaft thickness gain (+41%, *p* = 0.039) and vellus reduction (−36%, *p* = 0.025) were sustained, while the transient coverage gain at 3–4 months (+38%, *p* = 0.007) partially receded. **Conclusions**: In this preliminary case series, the integrative procedure was associated with quantifiable trichoscopic re-thickening rather than gross densification, with sustained shaft-caliber gain at four years in the long-term case. Causal attribution to any single component is not possible from this single-arm design; prospective controlled trials are required.

## 1. Introduction

Androgenetic alopecia (AGA) is the most common cause of progressive hair loss, with a lifetime prevalence approaching 80% in men and 40% in women of European ancestry, and lower but substantial prevalence in East Asian populations [1,2,3]. The condition is characterized by progressive follicular miniaturization (the conversion of large, pigmented terminal hairs into short, thin, depigmented vellus hairs), concentrated in the frontal and vertex scalp while the occipital and temporal regions are relatively spared [4]. Although the visible endpoint is loss of cosmetic hair coverage, the underlying process is a graded reduction in shaft caliber that can be quantified on trichoscopy independently of, and often earlier than, gross density change [5,6].

Current pharmacologic standard of care comprises topical minoxidil, oral finasteride, and, in selected patients, oral dutasteride [7,8,9]. These agents slow disease progression and produce modest, dose-dependent gains in hair count and shaft caliber, but their effect is partial and is sustained only with indefinite use; in long-term studies a substantial minority of patients exhibit limited or no response, and discontinuation typically reverses gains within 6–12 months [10,11]. Hair-transplant surgery achieves durable coverage in carefully selected patients but redistributes existing terminal follicles rather than increasing total follicular density [12]. The clinical reality, therefore, is that a meaningful proportion of patients with AGA continue to lose hair despite optimized pharmacotherapy and are unwilling or ineligible for transplant.

To address this gap, a heterogeneous class of adjuvant and combination procedures has emerged. Autologous platelet-rich plasma (PRP) injection improves shaft thickness and density across multiple randomized trials, with median effect sizes that are comparable to topical minoxidil [13,14]. Adipose-derived stromal vascular fraction (SVF) and adipose-derived stem-cell conditioned media show promising early efficacy, particularly when combined with PRP [15,16]. Intramuscular or intradermal botulinum toxin A (BTX) injection has been proposed to relieve scalp muscular tension and improve perfusion to the alopecic scalp, with preliminary clinical evidence of hair-count gains [17,18]. Each of these therapies has a plausible mechanism and a published evidence base; what remains unknown is whether sequential combination, within a single session and with adjunctive surgical components, produces additive, synergistic, or merely substitutive effects relative to the individual modalities.

A complementary line of reasoning has implicated chronic scalp tension and local microvascular compromise as contributory rather than incidental features of pattern hair loss [19,20]. Mechanically, the dense galea aponeurotica and the underlying scalp muscles transmit baseline tension that may, over time, restrict capillary perfusion at the follicular base. These observations have motivated procedural strategies that explicitly target scalp biomechanics and perfusion in addition to follicular pharmacology.

Building on this rationale, an integrative single-session multimodal scalp procedure has been used in routine clinical practice for more than five years, combining (i) partial temporalis muscle resection with silicone implantation, (ii) negative-pressure scalp stimulation, and (iii) BTX, PRP, and SVF injection. In the present study, we retrospectively quantified the trichoscopic outcomes of this procedure in a consecutively treated case series, using a fully automated image-analysis pipeline that yields six paired pre- and post-procedure metrics of shaft caliber, caliber heterogeneity, terminal-vellus distribution, and hair coverage. A subset of patients was followed for up to four years, allowing an exploratory examination of the long-term durability of the response. The aim was not to demonstrate efficacy against any specific comparator (the design does not permit such an inference), but rather to provide an objective, reproducible trichoscopic description of the response observed in routine practice, and to identify the components of the response that merit prospective controlled investigation.

## 2. Materials and Methods

### 2.1. Study Design and Patients

This was a single-center retrospective case series of patients with androgenetic alopecia (AGA) who underwent an integrative single-session multimodal procedure between 2020 and 2025 at Oakwood Bom Clinic, Seoul, Republic of Korea. The retrospective analysis presented in this manuscript was independently reviewed and approved by the Institutional Review Board of Ajou University Hospital (see Institutional Review Board Statement). The clinical database included 51 consecutively treated patients (34 men, 17 women); 28 patients (12 men, 16 women) completed a minimum clinical follow-up of four months and constituted the clinical-outcomes cohort. The mean age of the clinical-outcomes cohort was 54.5 years (range, 31–79 years), and 25 patients (89.3%) had received antiandrogen therapy (oral finasteride or dutasteride, or topical minoxidil) prior to the index procedure without sustained response.

**Figure 1:**
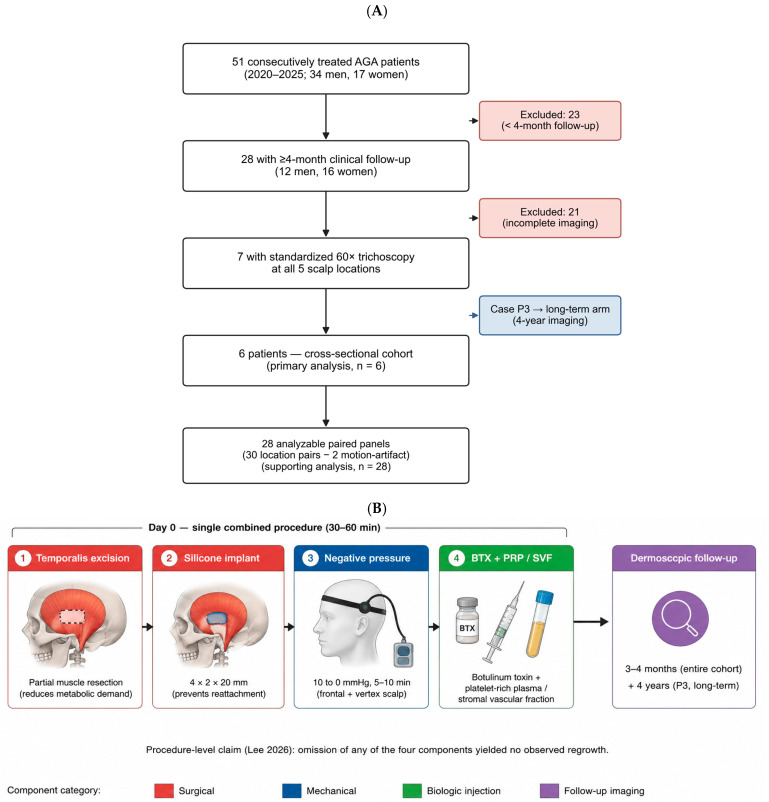
Study design: (**A**) Participant flow. From 51 consecutively treated patients with androgenetic alopecia (2020–2025), 28 completed at least four months of clinical follow-up; 7 had standardized 60× videodermoscopy at all five predefined scalp locations at both baseline and 3–4 months. Six formed the cross-sectional quantitative cohort (primary patient-level analysis, *n* = 6; 30 location pairs, of which 28 were analyzable after excluding two motion-artifact pairs, forming the supporting panel-level analysis, *n* = 28), and one additional patient (Case P3) had four-year imaging and was analyzed separately as an illustrative long-term case. (**B**) Treatment protocol. Anatomic schematic of the operative field (partial excision of the temporalis muscle, red shading; placement of a 4 × 2 × 20 mm silicone implant, gray) and treatment timeline: (1) partial temporalis muscle excision; (2) silicone implant placement to prevent muscle reattachment; (3) negative-pressure scalp stimulation applied to the frontal and vertex regions for 5–10 min with pressure descending from 10 mmHg to 0 mmHg; and (4) intramuscular and intradermal injections of botulinum toxin combined with autologous platelet-rich plasma (PRP) or stromal vascular fraction (SVF). Dermoscopic follow-up was performed at 3–4 months in the entire cohort and additionally at 4 years in Case P3.

For the present quantitative imaging analysis, a subset of patients was identified retrospectively from the clinical archive based on the availability of standardized 60× videodermoscopy at five predefined scalp locations performed at both baseline and follow-up. Seven patients met this criterion: six patients with paired baseline and 3–4-month follow-up imaging at all five locations (the quantitative cross-sectional cohort, hereafter “*n* = 6 patients; 30 location pairs, of which 28 were analyzable after excluding two motion-artifact pairs”), and one additional patient (Case P3) for whom a third imaging session at four years was also available (the long-term follow-up case). The discrepancy between the larger clinical cohort and the quantitative imaging subset is attributable to the retrospective nature of the analysis: standardized five-location videodermoscopy was not performed uniformly during the original clinical care, and only patients with complete, anatomically matched paired images were eligible for quantification. The derivation of the analyzed cohorts is summarized in Figure 1.

The inclusion criteria were (i) clinical diagnosis of male- or female-pattern AGA; (ii) age ≥ 18 years; (iii) availability of paired pre- and post-procedure videodermoscopic images at standardized anatomic locations. The exclusion criteria were (i) prior hair-transplant surgery; (ii) active scalp inflammation, infection, or scarring alopecia; (iii) systemic antiandrogen therapy initiated within four weeks of the index procedure.

### 2.2. Integrative Multimodal Procedure

All the procedures were performed in a single session of 30–60 min under monitored anesthesia care, combining intravenous sedation with local infiltration anesthesia, supervised by a board-certified anesthesiologist. The procedure comprised four sequential components delivered bilaterally:

(1) Partial temporalis muscle resection. Using the helical root of the ear as a reference, a 2.5 cm horizontal incision was made 2 cm anterior and 2 cm superior to the reference point. A segment of the temporalis muscle measuring approximately 20 × 10 mm was excised en bloc. The operator’s stated a priori rationale was reduction in metabolic demand in the temporoparietal region, with the aim of favoring redistribution of regional perfusion toward the frontoparietal scalp; as discussed in Section 4.2, this assumption is not established by classical vascular anatomy and is presented here as the operator’s hypothesis rather than as a demonstrated mechanism.

(2) Silicone implant placement. To prevent secondary re-adhesion of the resected muscle bed, a medical-grade silicone block (4 × 2 × 20 mm; SILASTIC™, Dow Corning, Midland, MI, USA) was inserted into the resection cavity prior to wound closure.

(3) Negative-pressure scalp stimulation. Following wound closure, a polyurethane foam dressing was placed along the suture line and connected to a negative-pressure wound therapy unit (V.A.C.® Therapy; KCI, San Antonio, TX, USA). Negative pressure was applied at the frontal and vertex scalp in a descending sequence from 10 mmHg to 0 mmHg over 5–10 min per side.

(4) Pharmacologic and biologic adjuvant injection. Botulinum toxin A (Botox; Allergan, Irvine, CA, USA) was administered intramuscularly into the temporalis muscle at 4 units per 1 × 1 cm grid. Stromal vascular fraction (SVF) was harvested by liposuction from the abdomen using the Duocell^®^ SVF kit (REV-MED Co., Ltd., Seongnam, Republic of Korea), yielding approximately 5 cc of SVF per side. The harvested SVF, alone or combined with autologous platelet-rich plasma (PRP) at the operator’s discretion, was injected into the subcutaneous layer overlying the alopecic frontoparietal regions. SVF was administered to all the patients, whereas the addition of PRP was at the operator’s discretion rather than by a fixed protocol.

Steps (1)–(4) were performed symmetrically on the contralateral side in the same session. Postoperative care consisted of routine surgical wound care; aside from the negative-pressure foam dressing applied along the suture line (component 3), no standardized scalp- or hair-specific aftercare regimen was prescribed, and no topical or systemic hair-growth pharmacotherapy was used during the postoperative follow-up period. An anatomic schematic of the operative field and the integrated treatment timeline is shown in Figure 1.

### 2.3. Image Acquisition

Trichoscopic imaging was performed using a 60× video dermoscope (Dino-Lite TrichoScope, AnMo Electronics Corp., New Taipei City, Taiwan) at five predefined scalp locations per patient: L1, intersection of the facial midline and the frontal hairline; L2, vertex; L3, midpoint between the frontal hairline and the vertex along the midline; L4, frontal hairline aligned with the left pupil; L5, frontal hairline aligned with the right pupil. Images were acquired at baseline (within four weeks prior to the procedure) and at the 3–4-month follow-up visit, with an additional acquisition at four years post-procedure for Case P3. Anatomic re-localization at follow-up was performed visually using each patient’s hairline contour and intrinsic scalp landmarks; a positional deviation of 3–5 mm between sessions was permitted by the original clinical protocol.

### 2.4. Quantitative Image Analysis

A standardized image-analysis pipeline was implemented in Python 3 (version 3.11) using the scikit-image (version 0.26.0), NumPy (version 2.3.0), SciPy (version 1.17.1), and Matplotlib (version 3.10.3) libraries. The pipeline was implemented with the assistance of a generative-AI coding tool (Claude Code, Anthropic; see Acknowledgments), with all code reviewed, executed, and validated by the authors. The full analysis protocol is described in the Supplementary Materials (File S1); the reference implementation is available from the corresponding author on reasonable request.

Calibration. The 60× video dermoscope had a nominal horizontal field of view of 3.0 mm. For each captured panel, the pixel-to-physical conversion was computed as μm per pixel = 3000/panel width in pixels. Because all the outcome metrics were within-image paired comparisons (baseline vs. follow-up acquired with the identical device on the same patient), absolute calibration error did not propagate into the paired effect estimates.

Region of interest (ROI). Each composite image was split horizontally at the inter-panel gray border (or, where absent, at the 50% midline) into a baseline panel and a follow-up panel. The ROI for analysis was defined as the central 80% of each panel (10% trimmed margin on each side) to exclude vignetting and edge artifacts. ROI dimensions and position were identical for paired baseline and follow-up panels.

Preprocessing. Color images were converted to luminance grayscale (Y = 0.299·R + 0.587·G + 0.114·B). Contrast-limited adaptive histogram equalization (CLAHE; clip limit 2.0, tile size 8 × 8) was applied to normalize illumination across panels, followed by a 3-pixel median filter to suppress sensor noise while preserving shaft edges.

Hair segmentation. Otsu’s automatic thresholding was applied to the preprocessed grayscale ROI; pixels darker than the threshold were classified as hair. The binary mask was cleaned by morphological opening with a 1-pixel disk to remove isolated speckles and by removing connected components smaller than 50 pixels.

Skeletonization and per-shaft thickness. Hair skeletons were obtained from the binary mask, and per-pixel shaft thickness was computed along the skeleton from the Euclidean distance transform of the mask. Per-pixel thickness was converted to μm using the per-image calibration.

Outcome metrics. Six quantitative outcomes were derived per panel: (i) median shaft thickness (μm); (ii) mean shaft thickness (μm); (iii) diameter heterogeneity, expressed as the coefficient of variation in per-pixel thickness (CV%); (iv) terminal-hair proportion: fraction of skeleton points with thickness > 50 μm (%); (v) vellus-hair proportion: fraction of skeleton points with thickness < 30 μm (%); (vi) hair coverage: hair-pixel area as a fraction of ROI area (%). The 30 μm and 50 μm thresholds for vellus and terminal hair classification follow the trichoscopy diameter conventions of Rakowska et al. [5]. Because these proportions are computed over skeleton pixels rather than over individually segmented hair shafts, the terminal- and vellus-hair proportions reported here are image-based pixel fractions and are not equivalent to per-follicle terminal and vellus counts obtained by manual trichoscopic grading.

A blinded reviewer with no role in the original clinical care executed the pipeline; no manual per-image ROI adjustment was permitted in order to limit selection bias. Quality-assurance overlays superimposing the segmentation mask on each panel were generated for visual inspection.

### 2.5. Statistical Analysis

Because the five anatomic locations imaged within each patient are not statistically independent, the primary analysis was performed at the patient level (*n* = 6 patients): each of the six outcome metrics was first averaged across the five anatomic locations within a patient, and within-patient change scores (Δ = follow-up–baseline) were then compared across the six patients. Treating the 28 individual panels as independent observations would overstate the effective sample size and understate the standard errors; the patient-level analysis is therefore adopted as the primary and more conservative inference, while the panel-level analysis (*n* = 28 paired panels from 6 patients across 5 anatomic locations) is reported as a higher-resolution supporting (sensitivity) analysis that corroborates it. A linear mixed-effects model with a patient-level random effect was considered but not adopted, because variance-component estimation is unreliable with only six clusters; the patient-averaged paired comparison is the more stable and conservative choice. For each metric and at both levels, change scores were tested for departure from normality using the Shapiro–Wilk test. Where the change-score distribution was consistent with normality (*p* > 0.05 in all six metrics in the present dataset), paired Student’s *t*-tests were applied; otherwise, the Wilcoxon signed-rank test was used as the fallback. Effect sizes were reported as Cohen’s d_n_ for paired data.

The four-year long-term case (P3) was analyzed separately at the within-patient level across the five anatomic locations and three time points (baseline, 3–4 months, and four years).

All the statistical tests were two-sided with α = 0.05. Continuous data are summarized as mean ± standard deviation, and percent change is reported relative to the baseline mean. Analyses were performed in Python 3.11 using SciPy version 1.17.1 and Pandas version 2.3.0.

## 3. Results

### 3.1. Patient Characteristics

Between 2020 and 2025, 51 patients (34 men, 17 women) underwent the integrative multimodal procedure. Twenty-eight patients (12 men, 16 women) completed a minimum clinical follow-up of four months and constituted the clinical-outcomes cohort (mean age 54.5 years, range 31–79). Twenty-five of these patients (89.3%) had received prior antiandrogen therapy without a sustained response. Fifteen patients (53.6%) were followed for at least twelve months; one patient (Case P3, female, age at procedure 31 years) was followed for four years. Formal Norwood–Hamilton or Ludwig severity grading was not uniformly recorded in the retrospective clinical records; baseline disease severity in the quantitative cohort is instead characterized objectively by the pre-treatment trichoscopic values (terminal-hair proportion 31.6 ± 10.6%, vellus-hair proportion 48.4 ± 10.8%, median shaft thickness 32.2 ± 9.3 μm; panel-level values, Table 1), consistent with established androgenetic alopecia.

Seven patients had standardized 60× videodermoscopy archived at all five predefined anatomic locations at both the baseline and 3–4-month time points and were therefore eligible for the quantitative trichoscopy analysis. Six of these constituted the cross-sectional quantitative cohort (*n* = 6 patients, 30 location-specific imaging pairs, of which 28 yielded analyzable paired panels after exclusion of two pairs with motion artifact). The seventh patient (Case P3) had an additional four-year imaging session and was analyzed separately as the long-term follow-up case (Section 3.4).

### 3.2. Panel-Level Analysis (Supporting; n = 28 Paired Panels)

**Figure 2:**
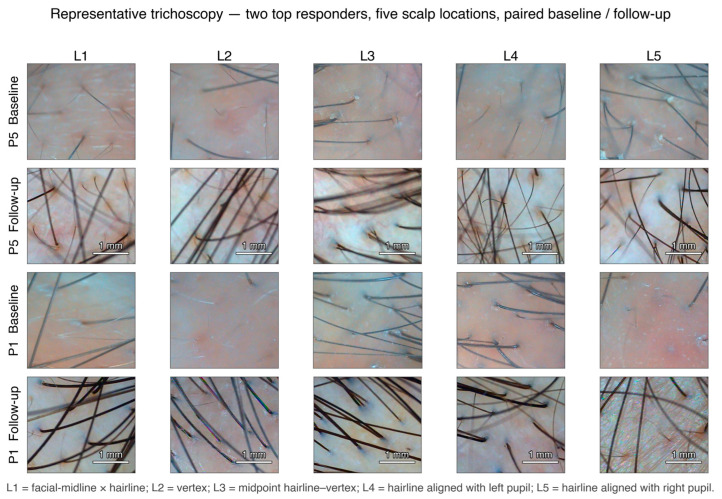
Representative trichoscopy: two top responders across five scalp locations. Paired baseline and follow-up trichoscopic images obtained at 60× magnification (horizontal field of view ≈ 3.0 mm; 1 mm scale bar at lower right of each follow-up panel) for the two patients with the largest improvement in median shaft thickness. For each patient, the top row shows the baseline image and the bottom row the follow-up image taken at 3–4 months. Each column corresponds to one of the five predefined scalp locations: L1 = intersection of facial midline and hairline; L2 = vertex; L3 = midpoint between hairline and vertex; L4 = hairline aligned with left pupil; L5 = hairline aligned with right pupil. Both patients show increased shaft density and caliber across all five locations, with conversion of fine vellus hairs (<30 μm) into thicker terminal hairs (>50 μm). Minor positional variation of 3–5 mm between sessions was permitted by protocol; the 80% central region of each panel was used for downstream quantification (Figure 3).

**Figure 3:**
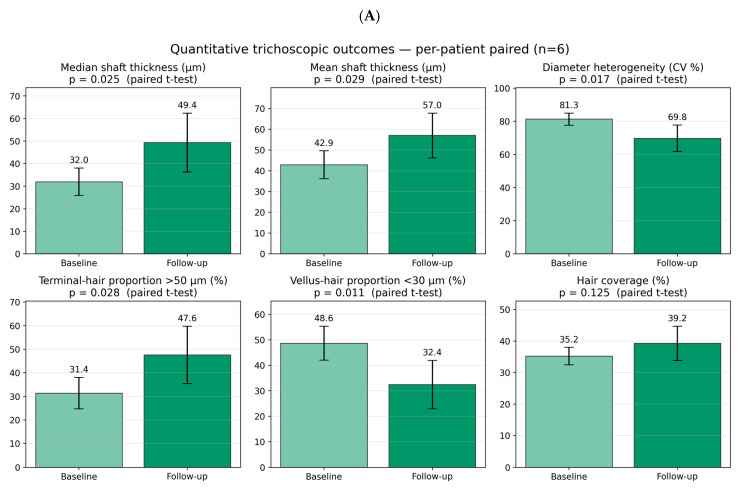

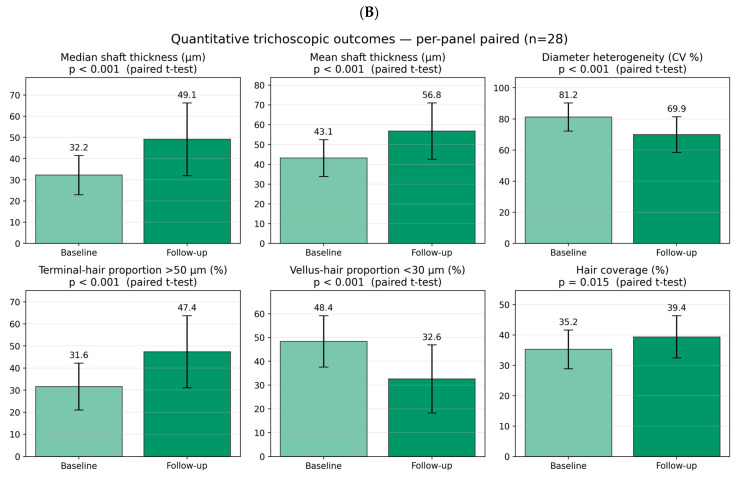
Quantitative trichoscopic outcomes: primary patient-level and supporting panel-level analyses: (**A**) Primary patient-level analysis (*n* = 6 patients; paired *t*-tests on patient-averaged values). Five of the six outcomes improved significantly (median shaft thickness, *p* = 0.025; mean shaft thickness, *p* = 0.029; diameter heterogeneity, *p* = 0.017; terminal-hair proportion, *p* = 0.028; vellus-hair proportion, *p* = 0.011), each with a large paired effect size (Cohen’s d_n_ = 1.23–1.63); hair coverage did not reach significance (*p* = 0.125; d_n_ = 0.75), consistent with a response of shaft re-thickening rather than gross densification. (**B**) Supporting panel-level sensitivity analysis (*n* = 28 paired panels). The smaller *p*-values in (**B**) arise because the five panels per patient are clustered and not independent, which inflates the effective sample size; the patient-level analysis in (**A**) is therefore the conservative, primary inference. Bars show the mean and error bars the standard deviation; *p*-values are from paired Student’s *t*-tests after Shapiro–Wilk normality testing of within-unit change scores (all *p* > 0.05).

This panel-level analysis resolves the response at the level of individual scalp locations and is reported as a higher-resolution, supporting analysis; the primary statistical inference, which accounts for the non-independence of the five locations within each patient, is presented at the patient level in Section 3.3. Representative paired baseline and 3–4-month follow-up trichoscopic images for the two patients with the largest gains in median shaft thickness are shown in Figure 2 across all five predefined scalp locations. The Shapiro–Wilk test did not reject normality for the within-panel change-score distribution of any of the six outcome metrics (*p* > 0.05 in all the cases), and paired Student’s *t*-tests were used as planned. All six metrics changed significantly between baseline and the 3–4-month follow-up (Figure 3B; Table 1). The complete set of paired baseline and follow-up trichoscopic images for all the imaged patients (35 paired panels across the five scalp locations) is provided in Supplementary File S3.

These panel-level *p*-values are smaller than those of the primary patient-level analysis because the within-patient locations are treated as independent (Section 2.5). The two principal indicators of shaft re-thickening (median shaft thickness and terminal-hair proportion) both increased by approximately 50% from baseline. Median shaft thickness rose from 32.2 ± 9.3 μm to 49.1 ± 17.2 μm (Δ = +16.9 ± 19.5 μm; +52.3%; t(27) = 4.57, *p* < 0.001; d_n_ = 0.86). Mean shaft thickness rose from 43.1 ± 9.3 μm to 56.8 ± 14.2 μm (Δ = +13.7 ± 16.5 μm; +31.7%; t(27) = 4.37, *p* < 0.001; d_n_ = 0.83). The terminal-hair proportion (>50 μm) rose from 31.6 ± 10.6% to 47.4 ± 16.3% (Δ = +15.7 ± 18.6%; +49.7%; t(27) = 4.48, *p* < 0.001; d_n_ = 0.85), while the vellus-hair proportion (<30 μm) declined from 48.4 ± 10.8% to 32.6 ± 14.3% (Δ = −15.8 ± 16.3%; −32.7%; t(27) = −5.13, *p* < 0.001; d_n_ = −0.97).

Diameter heterogeneity, measured as the coefficient of variation in per-pixel shaft thickness, decreased from 81.2 ± 9.1% to 69.9 ± 11.5% (Δ = −11.3 ± 13.7%; −13.9%; t(27) = −4.35, *p* < 0.001; d_n_ = −0.82), indicating that shafts became more uniform in caliber at follow-up. Hair coverage rose more modestly from 35.2 ± 6.4% to 39.3 ± 7.0% (Δ = +4.1 ± 8.4%; +11.7%; t(27) = 2.61, *p* = 0.015; d_n_ = 0.49); the change was statistically significant but the effect size was about half that of the thickness-based metrics, indicating that re-thickening of existing follicles, rather than gross densification, was the dominant trichoscopic signature of response. The full distribution of paired change scores across the six metrics is shown in Figure 3B.

### 3.3. Primary Patient-Level Analysis (n = 6 Patients)

The primary analysis was conducted at the patient level (*n* = 6 patients), averaging each of the six metrics across the five anatomic locations within each patient to respect the non-independence of within-patient locations. Five of the six metrics were significant at the patient level (median thickness, *p* = 0.025; mean thickness, *p* = 0.029; diameter heterogeneity, *p* = 0.017; terminal-hair proportion, *p* = 0.028; vellus-hair proportion, *p* = 0.011), each with a large paired effect size (|d_n_| = 1.23–1.63). Hair coverage increased but did not reach statistical significance at the patient level (Δ = +4.0 ± 5.4%; +11%; *p* = 0.125; d_n_ = 0.75), consistent with the smaller, predominantly shaft-caliber nature of the response and reinforcing that the dominant signature was re-thickening of existing follicles rather than gross densification. These patient-level (primary) results are shown in Figure 3A and Table 2.

Every patient improved on at least four of the six metrics. Response magnitude varied substantially across patients: Cases P1 (Δ median thickness +29.8 μm) and P5 (Δ +38.1 μm) showed the largest gains, whereas Case P4 was essentially flat on shaft-thickness and terminal-hair endpoints (Δ median thickness +3.6 μm; Δ terminal proportion −1.7%), and Case P7 showed a small decline in coverage (Δ −2.6%) despite improvement on the thickness metrics. No patient deteriorated on all six metrics, and no patient withdrew from follow-up because of adverse events.

**Table 2:** Primary patient-level analysis: quantitative trichoscopic outcomes between baseline and 3–4-month follow-up across six patients (each metric averaged over the five anatomic locations within a patient). Values are mean ± SD. Paired Student’s *t*-tests (df = 5); five of six outcomes were significant (*p* = 0.011–0.029) with large effect sizes (|d_n_| = 1.23–1.63), while hair coverage did not reach significance (*p* = 0.125). Effect sizes are Cohen’s d_n_ for paired data. This is the primary analysis; the panel-level analysis (Table 1) is reported as a supporting sensitivity analysis.

Metric	Baseline (Mean ± SD)	Follow-Up (Mean ± SD)	Δ (Mean ± SD)	% Change	t (df = 5)	*p*	d_n_
Median shaft thickness (μm)	32.0 ± 6.1	49.4 ± 13.1	+17.4 ± 13.4	+54.4%	3.17	0.025	1.30
Mean shaft thickness (μm)	42.9 ± 6.7	57.0 ± 10.8	+14.1 ± 11.4	+32.9%	3.02	0.029	1.23
Diameter heterogeneity (CV %)	81.3 ± 3.7	69.8 ± 8.1	−11.5 ± 8.0	−14.2%	−3.54	0.017	−1.44
Terminal-hair proportion > 50 μm (%)	31.4 ± 6.7	47.6 ± 12.1	+16.2 ± 13.0	+51.7%	3.06	0.028	1.25
Vellus-hair proportion < 30 μm (%)	48.6 ± 6.6	32.4 ± 9.5	−16.2 ± 10.0	−33.4%	−3.98	0.011	−1.63
Hair coverage (%)	35.2 ± 2.8	39.2 ± 5.4	+4.0 ± 5.4	+11.4%	1.84	0.125	0.75

### 3.4. Illustrative Four-Year Case (P3)

**Figure 4:**
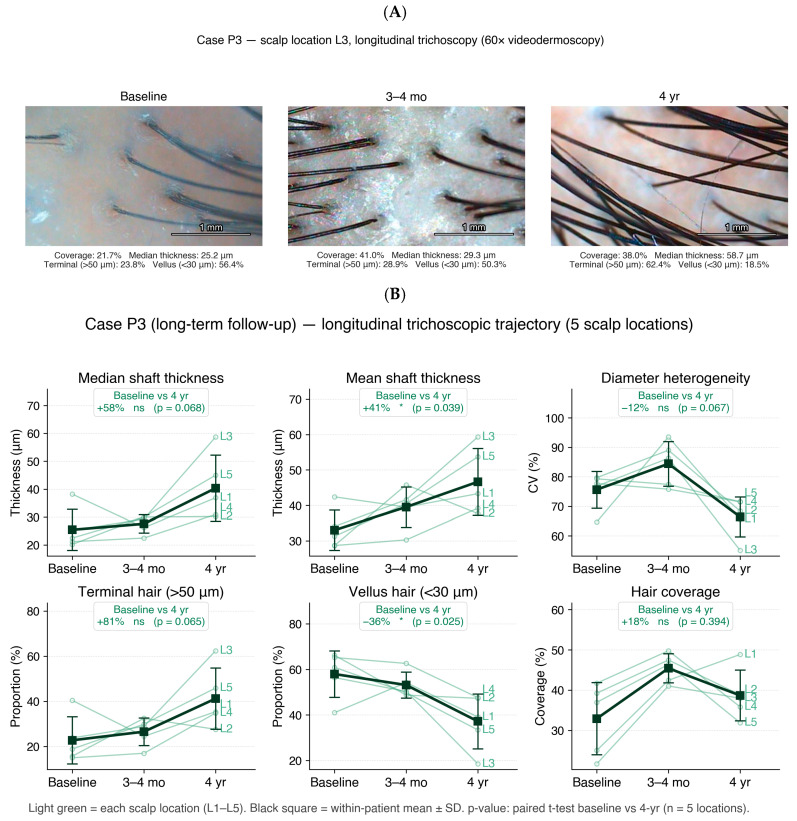
Long-term follow-up at four years (case P3). (**A**) Representative trichoscopic images at scalp location L3 (midpoint between hairline and vertex) for case P3 at baseline, 3–4 months, and 4 years following the combined procedure. L3 was selected because it showed the largest baseline-to-4-year increase in terminal-hair proportion (23.8% → 62.4%). At baseline, the field is dominated by fine vellus hairs and visible scalp; at 3–4 months, hair density has increased, but the shafts remain thin; at 4 years, fewer total hair shafts are visible, but those present are clearly thicker terminal hairs. Quantitative metrics for each panel are printed below the corresponding image. Each image was acquired at 60× magnification; 1 mm scale bars are shown at the lower right of each panel. (**B**) Quantitative trajectory of all six trichoscopic outcomes across the three time points for case P3, measured across all five scalp locations (L1–L5; light green lines). Black squares with error bars indicate the within-patient mean ± standard deviation. Asterisks (*) denote a statistically significant within-patient change versus baseline (paired *t*-test, *p* < 0.05); ns, not significant.

Case P3 (female, 31 years at the time of the procedure) had imaging at three time points: baseline, 3–4 months, and four years post-procedure (Figure 4). Because this four-year trajectory derives from a single patient (*n* = 1) imaged at five correlated locations, it is exploratory and hypothesis-generating. Across these five anatomic locations, mean shaft thickness rose from baseline to four years (Δ = +41%, *p* = 0.039), and the vellus-hair proportion decreased (Δ = −36%, *p* = 0.025); both effects were sustained at four years and were of similar magnitude to the cross-sectional cohort response at 3–4 months. The trajectory of hair coverage differed: coverage peaked transiently at 3–4 months (+38% vs. baseline, *p* = 0.007) and partially receded by four years (+18% vs. baseline, non-significant), whereas median shaft thickness and terminal-hair proportion continued to trend upward, and shaft-diameter heterogeneity continued to decrease, between the 3–4-month and four-year time points (Figure 4B); these trends did not reach significance at the per-location *n* = 5 sample size (median thickness *p* = 0.068; terminal proportion *p* = 0.065; shaft heterogeneity *p* = 0.067). At the representative location L3, which showed the largest baseline-to-four-year increase in terminal-hair proportion (23.8% → 62.4%), the qualitative trichoscopic appearance shifted from a field dominated by fine vellus hairs and visible scalp at baseline, to a denser field of still-thin shafts at 3–4 months, to a less densely populated but markedly thicker field at four years (Figure 4A). This dissociation between transient densification and sustained re-thickening is, to the authors’ knowledge, the first quantitative four-year trichoscopic documentation of a single case treated with this procedure; given the single-patient (*n* = 1) basis, this observation should be regarded as exploratory and hypothesis-generating rather than as evidence of durable efficacy.

### 3.5. Safety

Across the 51-patient clinical cohort, no serious complications (including infection, wound dehiscence, neuromuscular dysfunction, or aggravation of alopecia) were observed during follow-up. Transient mild edema, bruising, or petechiae at the incision site were noted in a minority of patients and resolved spontaneously without intervention. Postoperative discomfort was managed with non-steroidal anti-inflammatory drugs. Adverse events were monitored clinically at each follow-up visit; clinical follow-up extended to a minimum of four months in the outcomes cohort, to twelve months or more in 53.6% of patients, and to four years in the long-term case, and no delayed complications related to the temporalis resection or silicone implant were recorded over this period. No standardized adverse-event grading instrument was applied, which is acknowledged as a limitation of the retrospective safety assessment.

## 4. Discussion

### 4.1. Principal Findings

In the primary patient-level analysis (*n* = 6 patients), five of the six trichoscopic metrics improved significantly at 3–4 months, each with a large paired effect size (|d_n_| = 1.23–1.63): median shaft thickness rose by +54% and terminal-hair proportion by +52%, while vellus-hair proportion fell by 33% and shaft-diameter heterogeneity by 14%, indicating both larger and more uniform shafts at follow-up. Hair coverage increased but did not reach statistical significance (+11%, *p* = 0.125), reinforcing that the dominant response was re-thickening of existing follicles rather than gross densification. These changes were concordant with, and of similar magnitude to, the higher-resolution panel-level supporting analysis (*n* = 28 panels; |d_n_| = 0.49–0.97). In the single illustrative long-term case (P3; *n* = 1, exploratory), mean shaft thickness (+41%, *p* = 0.039) and reduced vellus proportion (−36%, *p* = 0.025) were sustained at four years, whereas the larger transient coverage gain at 3–4 months (+38%, *p* = 0.007) partially receded by year four.

### 4.2. Mechanism Interpretation

Each component has an independent biological rationale. PRP delivers concentrated platelet-derived growth factors (PDGF, VEGF, IGF-1) and has been shown to increase shaft caliber in randomized trials [13,14]. Adipose-derived SVF supplies stromal cells and angiogenic mediators that may promote dermal papilla recovery [15]. Intramuscular BTX may improve scalp perfusion by relieving compressive forces on the subgaleal microvasculature [17,18]. Partial temporalis resection with silicone interposition is the most mechanistically novel and least literature-supported component; the operator’s rationale is reduction in metabolic competition between the temporalis and overlying scalp tissue.

The perfusion rationale warrants candid evaluation. The frontoparietal scalp is supplied predominantly by the superficial temporal, supraorbital, supratrochlear, and occipital arteries, while the temporalis muscle is supplied by the deep temporal arteries [21,22]. Because the deep and superficial temporal circulations are anatomically distinct, reducing temporalis metabolic demand does not, on classical vascular anatomy alone, predict redistribution of arterial flow into the frontoparietal scalp. The observed re-thickening may therefore be driven primarily by the biologic and pharmacologic components, with the surgical components contributing through a different mechanism (for example, wound-healing signaling) or as a non-essential addition. The present design cannot distinguish among these possibilities.

### 4.3. Comparison with the Existing Literature

Response magnitudes here lie at the upper end of monotherapy reports. PRP randomized trials report 10–30% shaft-thickness gain and 20–35% terminal-hair-count gain at 3–6 months [13,14], compared with +33% mean shaft thickness and +52% terminal-hair proportion (a related but not directly equivalent metric) in the present cohort (patient-level primary analysis, *n* = 6). Adipose-derived SVF and conditioned-media trials report comparable but more heterogeneous gains [15,16]. BTX studies report approximately 18% hair-count gain at 24 weeks [17,18]. The negative-pressure component applied here (a descending sequence from 10 to 0 mmHg over 5–10 min per side) operates well below the magnitudes used in therapeutic negative-pressure wound therapy, and we are aware of no direct clinical evidence that such a brief, low-pressure single-session stimulus independently promotes hair growth; its contribution therefore remains hypothetical and cannot be isolated in this multimodal design. Combination-therapy gains tend to be additive rather than synergistic [23]. The present effect sizes are consistent with, though not conclusively in excess of, the sum of published monotherapy effects, so the marginal contribution of the surgical components remains an open question.

### 4.4. Limitations

Several structural features of the present study constrain the strength of any causal inference and should be considered before any clinical translation.

First, the single-arm, retrospective design without a concurrent control precludes attribution of the observed change to the procedure itself; spontaneous hair-cycle synchronization, regression to the mean, and unmeasured concurrent therapy could each contribute. Second, with four simultaneous procedural components, no individual component effect can be isolated. Third, all care, imaging, and outcome adjudication were performed by a single operator at a single private clinic; the absence of a blinded independent assessor is a recognized bias source. Fourth, the quantitative cohort (*n* = 6 patients, 28 panels) was substantially smaller than the parent clinical cohort (*n* = 28 of 51 enrolled) and was identified retrospectively from patients with the most complete archived imaging, which may correlate with better adherence and outcome. Fifth, absolute shaft-diameter values rely on a single calibration assumption (60× FOV of 3.0 mm); within-panel paired comparisons are unaffected, but absolute terminal and vellus thresholds are approximate. Sixth, the four-year analysis is a single patient at five locations; the trending but non-significant changes in median thickness, terminal proportion, and heterogeneity at four years should not be interpreted as proof of durability beyond the two metrics that reached significance. Seventh, retrospective ethics review and approval of the analysis presented here were obtained from the Institutional Review Board of Ajou University Hospital in 2025, after the index procedures had been performed; the institutional ethics oversight applicable to the procedural innovation itself at the time of original clinical care was therefore retrospective rather than prospective. Eighth, the multimodal procedure described here has not yet been independently replicated by operators or clinics other than that of the originating clinician, which limits external validity until such replication occurs. Finally, several patient-level variables were not systematically captured in the retrospective clinical records and therefore could not be analyzed or reported, including individual reasons for loss to follow-up, baseline Norwood–Hamilton/Ludwig severity grade, duration of alopecia, prior treatment history, and the specific biologic administered (PRP, SVF, or both) for each patient; in addition, the sex composition shifted from 67% men at enrollment (34/51) to 43% men in the ≥4-month follow-up cohort (12/28), indicating differential attrition by sex. These gaps, intrinsic to the retrospective design, further temper the external validity of the quantitative findings and should be addressed prospectively.

### 4.5. Future Directions

This quantitative description is hypothesis-generating, not evidence of efficacy. A defensible next step is a prospective, parallel-group randomized trial comparing the integrative procedure with minoxidil monotherapy or a sham-controlled subset, with blinded outcome adjudication and quantitative trichoscopy as a co-primary outcome. A component-dropout factorial design would, in principle, isolate each component’s contribution but may exceed single-center resources. The quantitative trichoscopy pipeline used here (Supplementary File S1) is low-cost and operator-independent and could be transplanted to such a trial without modification.

## 5. Conclusions

In this preliminary retrospective case series, a four-component single-session scalp procedure for androgenetic alopecia was associated with significant, large-effect trichoscopic shaft re-thickening at 3–4 months in the primary patient-level analysis (*n* = 6 patients), the dominant signature being increased shaft caliber and terminal-hair proportion rather than gross densification; hair coverage did not change significantly at the patient level. In a single illustrative case, shaft-caliber gains were sustained at four years. Because the design is single-arm, uncontrolled, single-operator, and based on a small, retrospectively selected imaging cohort, these findings cannot establish efficacy or attribute the response to any individual component and should be regarded as hypothesis-generating. They do, however, provide an objective, reproducible quantitative-trichoscopy framework and a rationale for prospective, controlled, blinded evaluation of the procedure.

## Figures and Tables

**Table 1:** Quantitative trichoscopic outcomes between baseline and 3–4-month follow-up across 28 paired scalp panels (six patients × five anatomic locations, after exclusion of two pairs with motion artifact). This panel-level analysis is reported in support of the primary patient-level analysis (Table 2; Section 3.3). Values are mean ± SD. Paired Student’s *t*-tests were applied after Shapiro–Wilk normality testing of within-panel change scores (all *p* > 0.05). Effect sizes are reported as Cohen’s d_n_ for paired data.

Metric	Baseline (Mean ± SD)	Follow-Up (Mean ± SD)	Δ (Mean ± SD)	% Change	t (df = 27)	*p*	d_n_
Median shaft thickness (μm)	32.2 ± 9.3	49.1 ± 17.2	+16.9 ± 19.5	+52.3%	4.57	<0.001	0.86
Mean shaft thickness (μm)	43.1 ± 9.3	56.8 ± 14.2	+13.7 ± 16.5	+31.7%	4.37	<0.001	0.83
Diameter heterogeneity (CV %)	81.2 ± 9.1	69.9 ± 11.5	−11.3 ± 13.7	−13.9%	−4.35	<0.001	−0.82
Terminal-hair proportion > 50 μm (%)	31.6 ± 10.6	47.4 ± 16.3	+15.7 ± 18.6	+49.7%	4.48	<0.001	0.85
Vellus-hair proportion < 30 μm (%)	48.4 ± 10.8	32.6 ± 14.3	−15.8 ± 16.3	−32.7%	−5.13	<0.001	−0.97
Hair coverage (%)	35.2 ± 6.4	39.3 ± 7.0	+4.1 ± 8.4	+11.7%	2.61	0.015	0.49

CV, coefficient of variation; SD, standard deviation; df, degrees of freedom; d_n_, Cohen’s d for paired data.

## Data Availability

The de-identified quantitative trichoscopic data analyzed in this study (per-panel CSVs, per-patient aggregates, and the long-term case data) are provided in Supplementary File S2. The de-identified trichoscopy images are provided in Supplementary File S3. The image-analysis reference implementation (source code) is available from the corresponding author on reasonable request. The original full-resolution videodermoscopic image files are not publicly archived due to patient-privacy considerations but may be made available, in de-identified form and subject to ethics-committee approval, from the corresponding author on reasonable request for the purpose of methodological replication.

## Supplementary Materials

The following supporting information can be downloaded at https://www.mdpi.com/article/10.3390/jcm15135055/s1, File S1, Supplementary Materials Description (DOCX, a guide to the supplementary package including sample ID mapping, worksheet contents, image-archive structure, and reproducibility notes); File S2, De-identified quantitative data (XLSX, six sheets: per-panel, per-patient, per-sample raw values, and case P3 long-term means and raw values); File S3, De-identified trichoscopy images (ZIP, 35 paired-panel PNGs organized by anonymized sample ID with inventory and README).
