# Supplementary material for: Novel Combination Scalp Therapy for Androgenetic Alopecia: A Preliminary Retrospective Case Series with an Illustrative Four-Year Case
Source: J Clin Med. 2026 Jun 29;15(13):5055. doi: 10.3390/jcm15135055 (PMC13362154; doi:10.3390/jcm15135055)
Supplement: Supplementary file 1 [file jcm-15-05055-s001.zip › Supplementary_File_S1_Description.pdf]

Supplementary File S1: Supplementary Materials Description

Companion to: Novel Combination Scalp Therapy for Androgenetic Alopecia: A Preliminary Retrospective Case Series with an Illustrative Four-Year Case

Author: Jong-Hee Lee MD, Hyung Min Hahn MD

This document describes the contents, organization, and intended use of the supplementary materials accompanying this manuscript. The image-acquisition, image-analysis, and statistical methodology themselves are described in Sections 2.3–2.5 of the main manuscript and are not duplicated here.

S1.1. Overview of the Supplementary Materials Package

Three supplementary files accompany this manuscript, all referenced from the Back Matter Supplementary Materials statement:

- File S1 (this DOCX): description of the supplementary materials and the de-identification scheme used across the package.
- File S2 (XLSX): de-identified quantitative trichoscopy data in six worksheets; the primary numerical source for Table 1 and Sections 3.2–3.4 of the main manuscript.
- File S3 (ZIP): de-identified trichoscopy image archive containing 35 composite videodermoscopy panels, organized by anonymized sample ID and accompanied by an inventory and a README.

All patient identifiers (name, date of birth, clinical encounter date, clinic identifier) have been removed from S2 and S3. Sample IDs (P1 through P7) are used consistently across the main manuscript text, S2 worksheets, and S3 folder names so that any value or image can be traced unambiguously between files without exposing identifying information.

S1.2. Sample ID Mapping

The seven sample IDs correspond to the cases cited in the main manuscript text as follows:

- P1: cross-sectional case, among the two largest gainers in median shaft thickness.
- P2: cross-sectional case.
- P3: long-term follow-up case, with additional imaging at four years (Section 3.4 of the main manuscript).
- P4: cross-sectional case, essentially flat on shaft-thickness endpoints.
- P5: cross-sectional case, among the two largest gainers in median shaft thickness.
- P6: cross-sectional case.
- P7: cross-sectional case, with a small decline in hair coverage despite gains in thickness metrics.

S1.3. File S2 Description (Quantitative Data, XLSX)

The Excel workbook contains six worksheets. All numeric values were generated by the image-analysis pipeline described in main-manuscript Section 2.4 and the statistical analysis described in Section 2.5.

- README: provenance, sheet index, and sample ID mapping (this same information is reproduced in this S1 document).
- S2.1_Panel_paired_n28: per-panel paired statistics across 28 paired baseline / 3–4-month panels (6 samples × ~5 locations). One row per metric; columns include baseline mean ± SD, follow-up mean ± SD, paired t-statistic, p-value, and Cohen's d_z. This is the numerical source for Table 1 of the main manuscript.
- S2.2_Patient_paired_n6: patient-level paired statistics. The six metrics were first averaged across the five anatomic locations within each of the six cross-sectional samples, and paired t-tests were applied across the six per-sample means.
- S2.3_Per_patient_metrics: per-sample baseline and 3–4-month values for the six trichoscopic outcomes, listed one row per sample (P1, P2, P4–P7). This is the source table for the per-sample response heterogeneity described in Section 3.3.
- S2.4_P3_longterm_means: mean ± SD across the five anatomic locations within sample P3 at each of the three time points (baseline, 3–4 months, four years). This is the source for the long-term values reported in Section 3.4 and the trajectory plot in Figure 4B.
- S2.5_P3_longterm_raw: raw per-location values for sample P3 at the three time points (5 locations × 3 timepoints = 15 rows), provided so that the means and paired tests in S2.4 can be re-derived independently if a reviewer wishes to do so.

S1.4. File S3 Description (Trichoscopy Image Archive, ZIP)

The ZIP archive contains 35 de-identified composite trichoscopy panels organized in the following folder structure:

```
S3_images/
├── README.txt
├── inventory.csv
├── P1/
│   ├── loc1.png
│   ├── loc2.png
│   ├── loc3.png
│   ├── loc4.png
│   └── loc5.png
├── P2/  (loc1.png … loc5.png)
├── P3/  (loc1.png … loc5.png, 3-panel composites)
├── P4/  (loc1.png … loc5.png)
├── P5/  (loc1.png … loc5.png)
├── P6/  (loc1.png … loc5.png)
└── P7/  (loc1.png … loc5.png)
```

Each PNG is a vertically concatenated composite panel produced during routine clinical imaging:

- Samples P1, P2, P4, P5, P6, P7: 2-panel composite (top = baseline; bottom = 3–4-month follow-up).
- Sample P3: 3-panel composite (top = baseline; middle = 3–4-month follow-up; bottom = 4-year follow-up).

Image format: 60× video dermoscopy; horizontal field of view ≈ 3.0 mm per panel; PNG. Location naming L1–L5 follows the five predefined anatomic locations defined in main-manuscript Section 2.3 (L1 = midline-hairline intersection; L2 = vertex; L3 = midpoint between hairline and vertex; L4, L5 = hairline aligned with left and right pupil, respectively).

Two paired images (out of an arithmetic total of 30 cross-sectional pairs from six samples × five locations) were excluded from quantitative analysis due to motion artifact, leaving 28 analyzable paired panels for the n = 28 panel-level statistics. The excluded pairs are nonetheless included in the archive so that this exclusion can be independently audited; the inventory.csv inside the archive flags which panels were carried forward.

S1.5. Inventory CSV

S3_images/inventory.csv inside the archive provides the per-image mapping with columns: sample_id, location, filename_in_zip, panel_structure (2-panel vs 3-panel), timeline, and free-text notes. The inventory contains zero patient names; sample IDs are the only subject identifier.

S1.6. Reproducibility

All image analysis was performed with Python 3.11 and the following libraries: scikit-image (preprocessing, segmentation, skeletonization), NumPy (array operations), SciPy (paired t-tests via scipy.stats.ttest_rel, normality testing via scipy.stats.shapiro), Pandas (data aggregation), and Matplotlib (figure rendering). Exact package versions used in the present analysis are reported in the manuscript Methods Section 2.5.

To re-derive the values in S2 from the images in S3, the pipeline summarized in main-manuscript Section 2.4 must be applied to each composite panel in S3 with the ROI and segmentation parameters specified there. The reference implementation used to produce S2 is available from the author on reasonable request, as stated in the Data Availability Statement of the main manuscript.

S1.7. De-Identification and Consent

Patient names, dates of birth, encounter dates, and clinic identifiers have been removed from all files in this supplementary package. The composite trichoscopy panels in S3 show scalp surface only and do not contain facial features; nevertheless, because individual patients could in principle be identified by a reader who already knows them, the images are treated as identifiable, and the Informed Consent Statement in the main-manuscript Back Matter applies to their inclusion.
