# Supplementary figures and images for: Novel Combination Scalp Therapy for Androgenetic Alopecia: A Preliminary Retrospective Case Series with an Illustrative Four-Year Case
Source: J Clin Med. 2026 Jun 29;15(13):5055. doi: 10.3390/jcm15135055 (PMC13362154; doi:10.3390/jcm15135055)

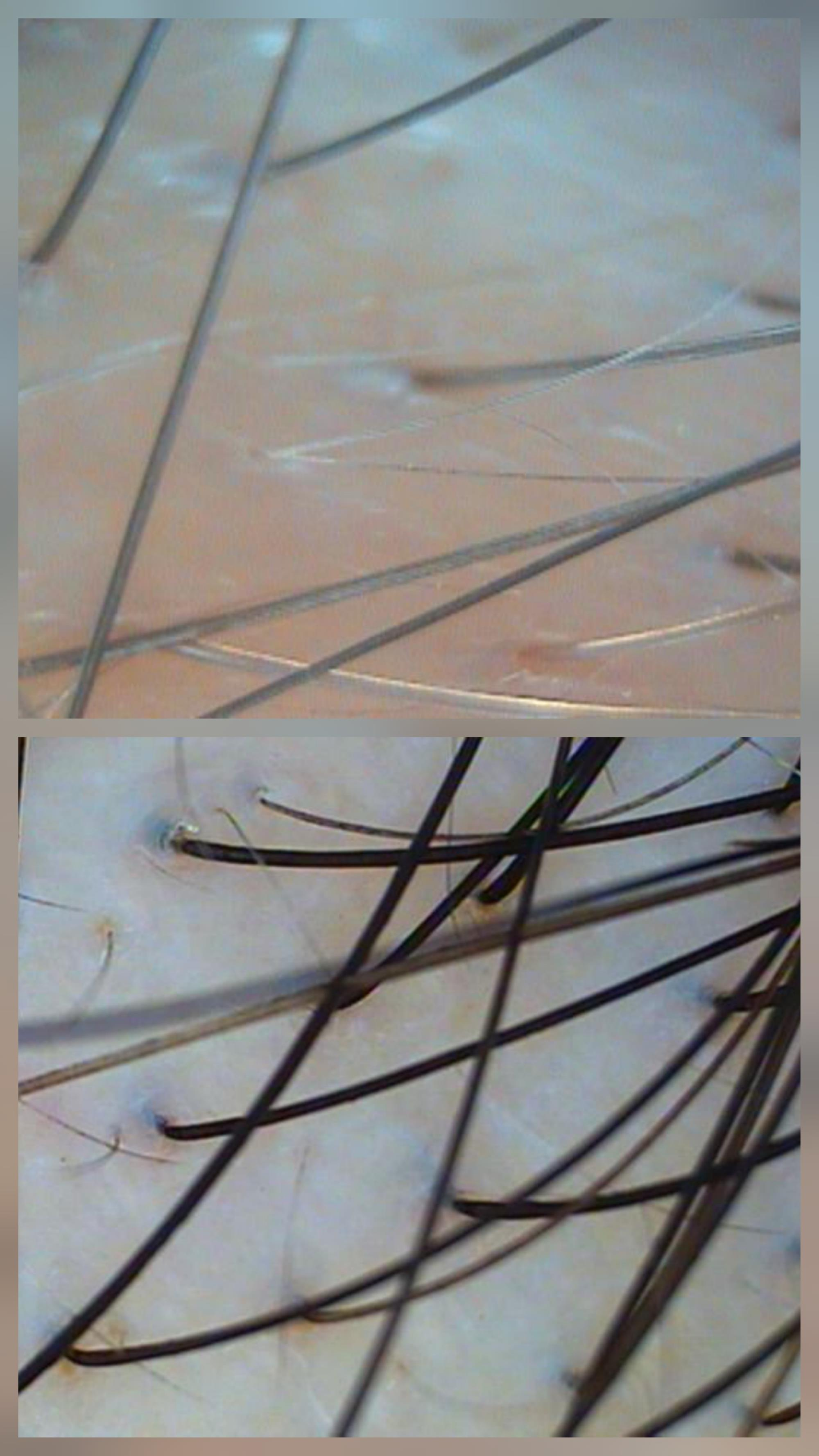

Supplement: Supplementary file 1 [file jcm-15-05055-s001.zip › Supplementary_File_S3_Trichoscopy_Images/S3_images/P1/loc1.png]

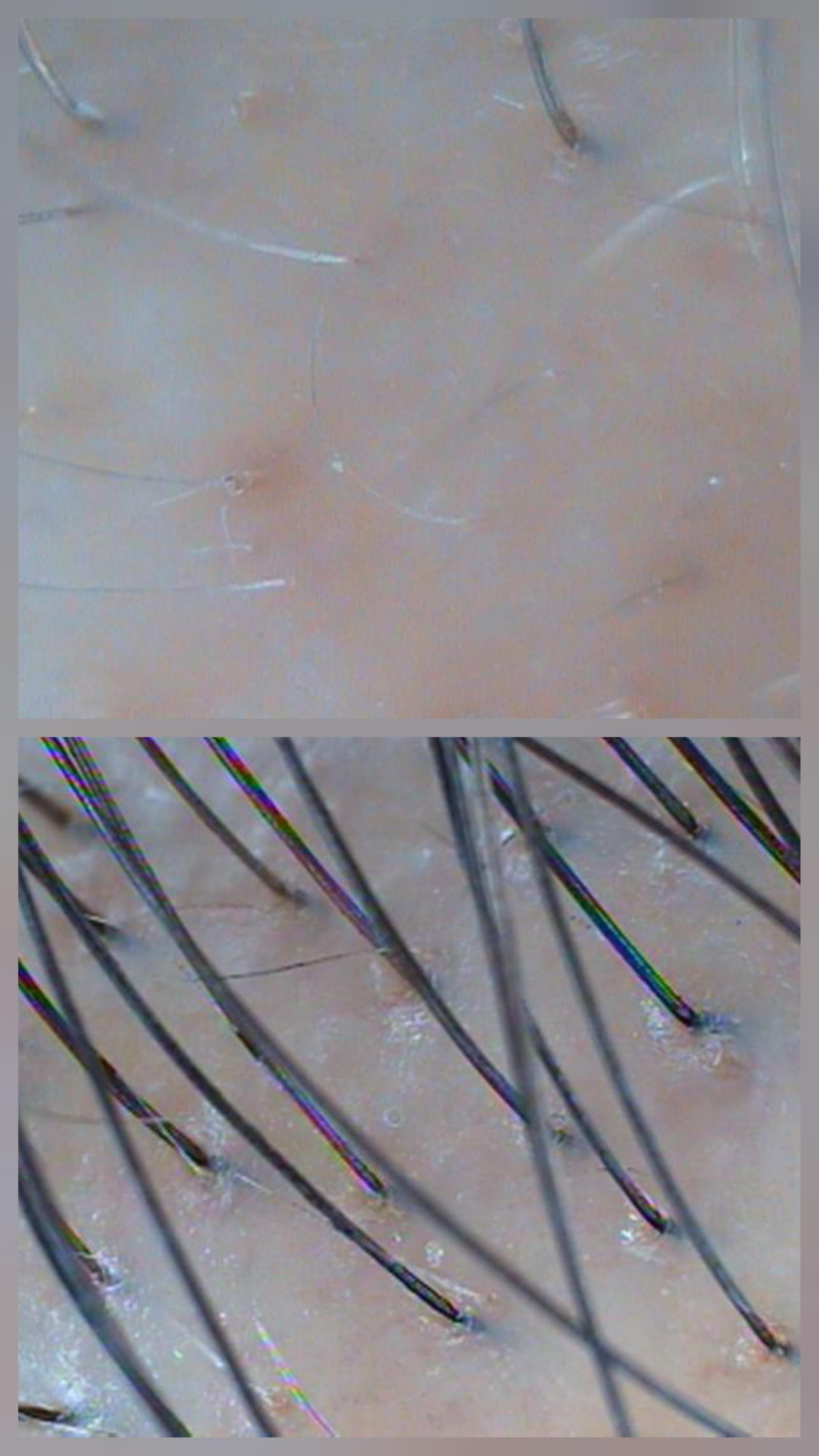

Supplement: Supplementary file 1 [file jcm-15-05055-s001.zip › Supplementary_File_S3_Trichoscopy_Images/S3_images/P1/loc2.png]

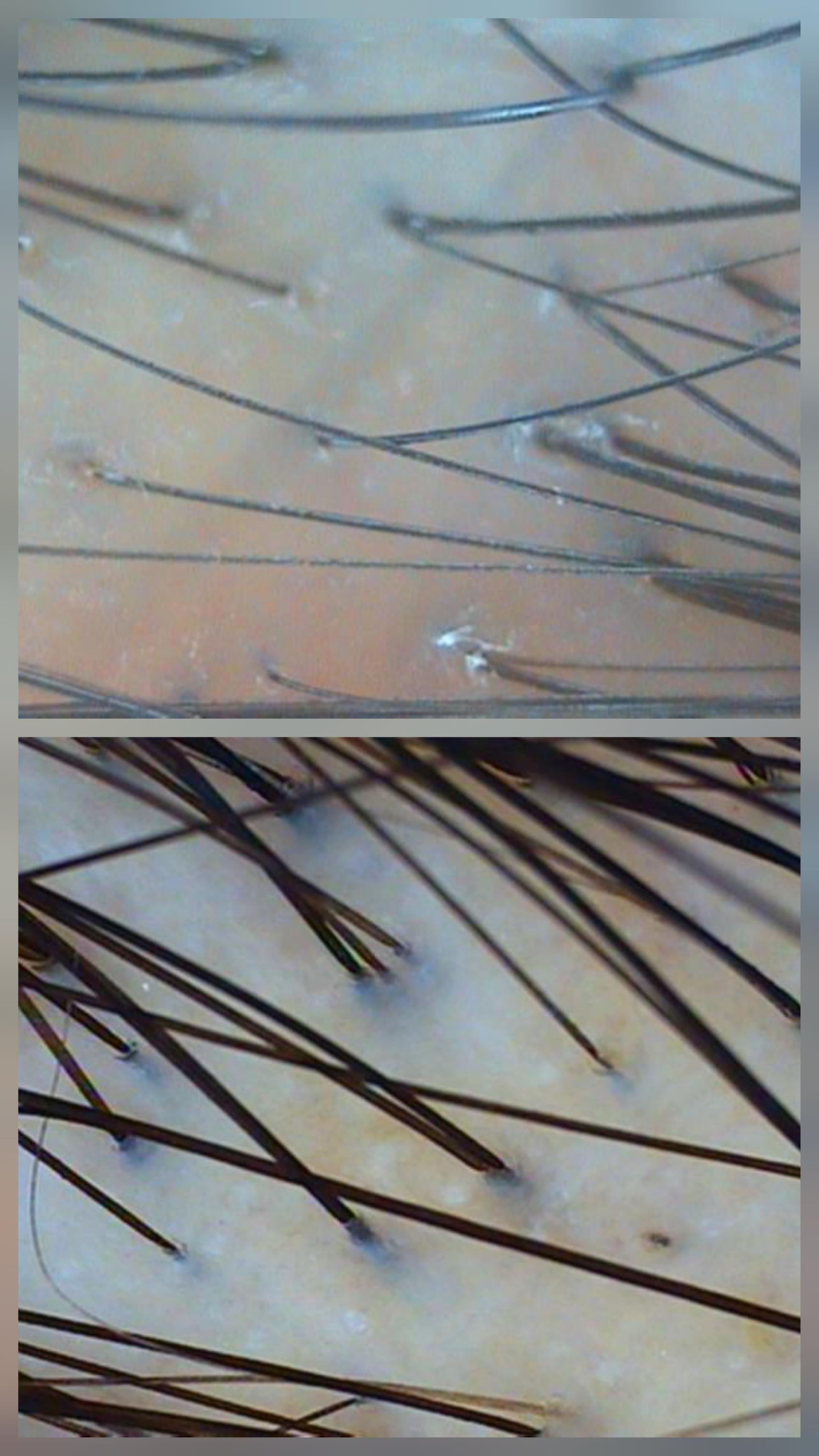

Supplement: Supplementary file 1 [file jcm-15-05055-s001.zip › Supplementary_File_S3_Trichoscopy_Images/S3_images/P1/loc3.png]

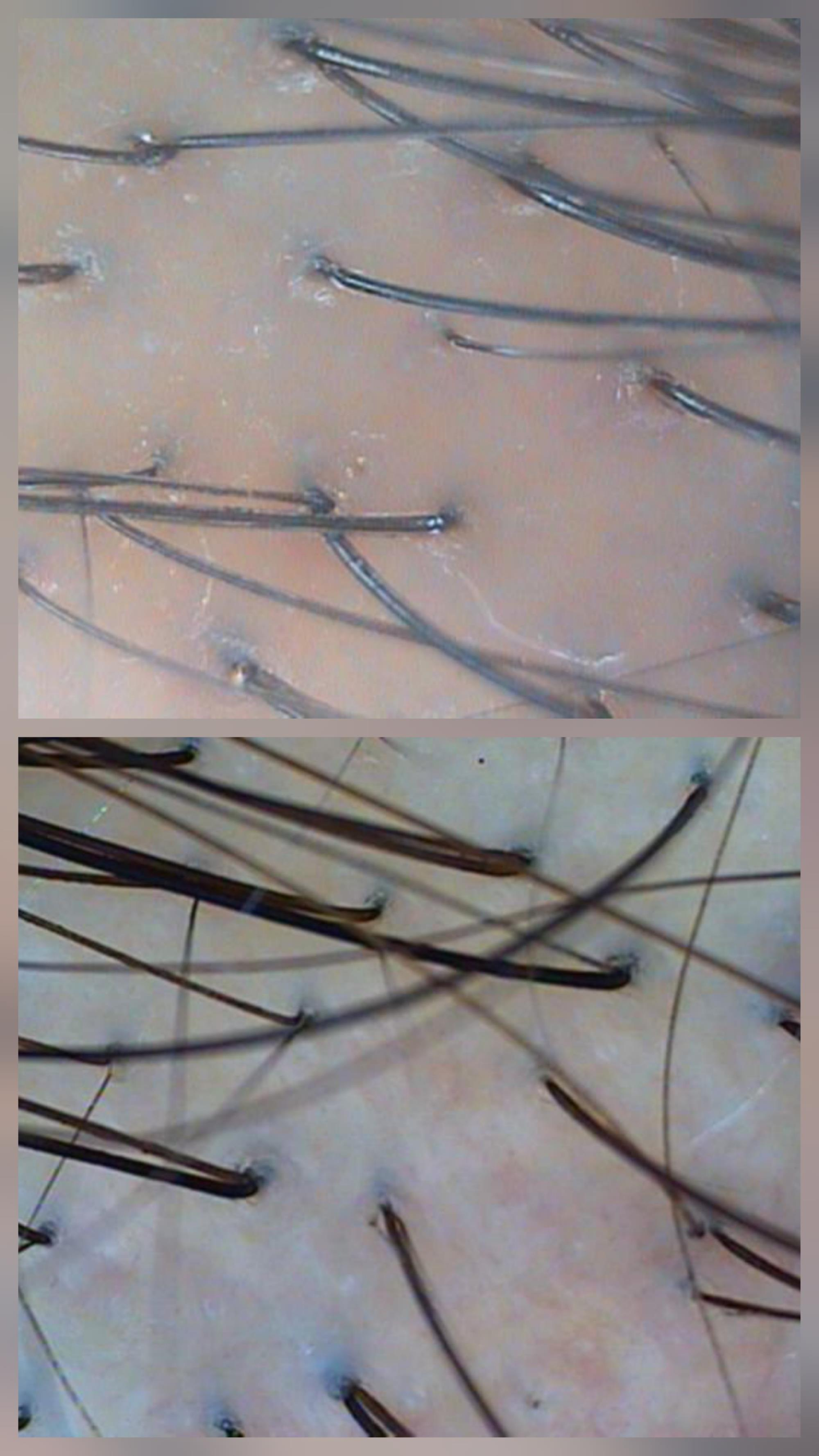

Supplement: Supplementary file 1 [file jcm-15-05055-s001.zip › Supplementary_File_S3_Trichoscopy_Images/S3_images/P1/loc4.png]

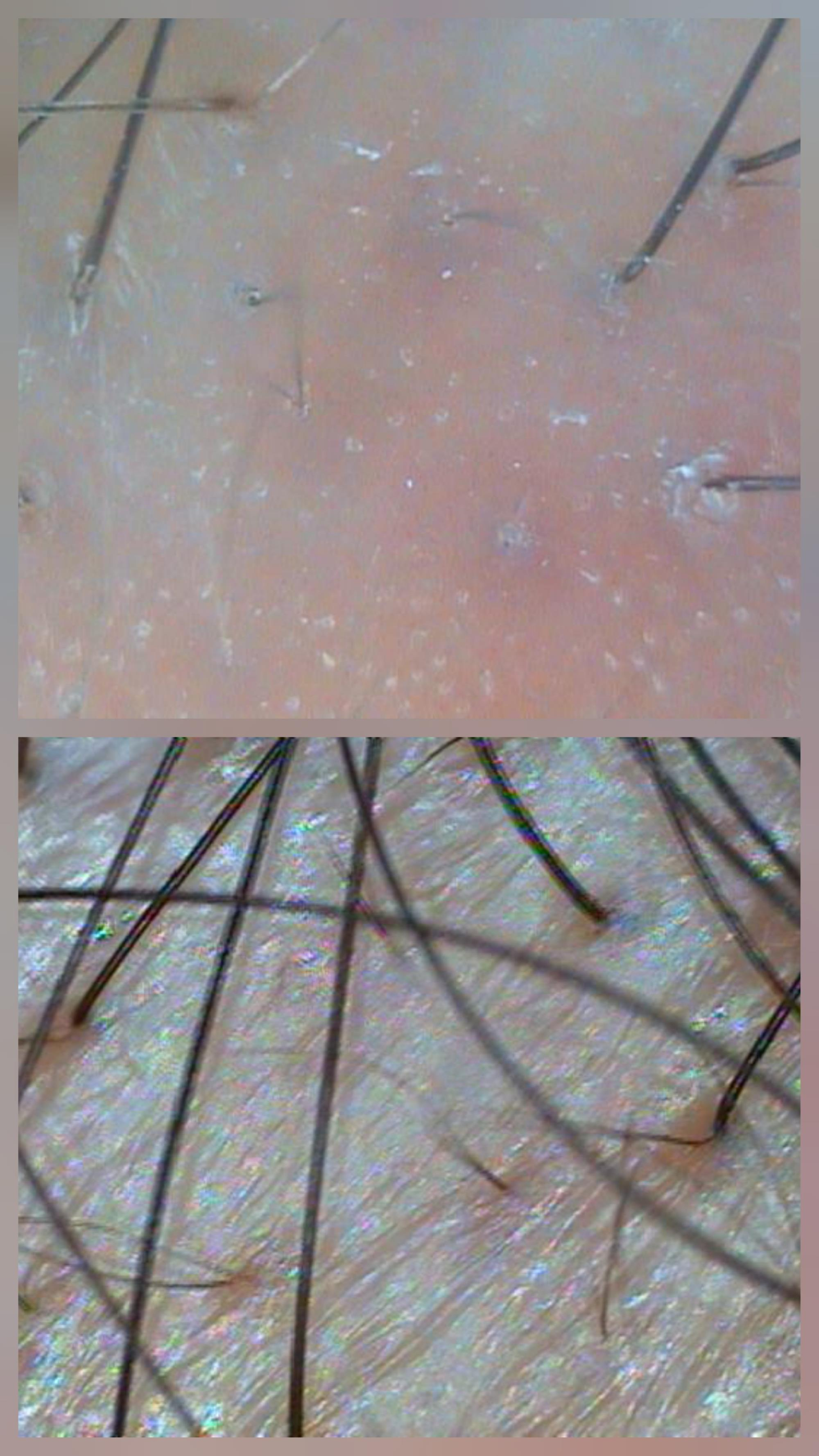

Supplement: Supplementary file 1 [file jcm-15-05055-s001.zip › Supplementary_File_S3_Trichoscopy_Images/S3_images/P1/loc5.png]

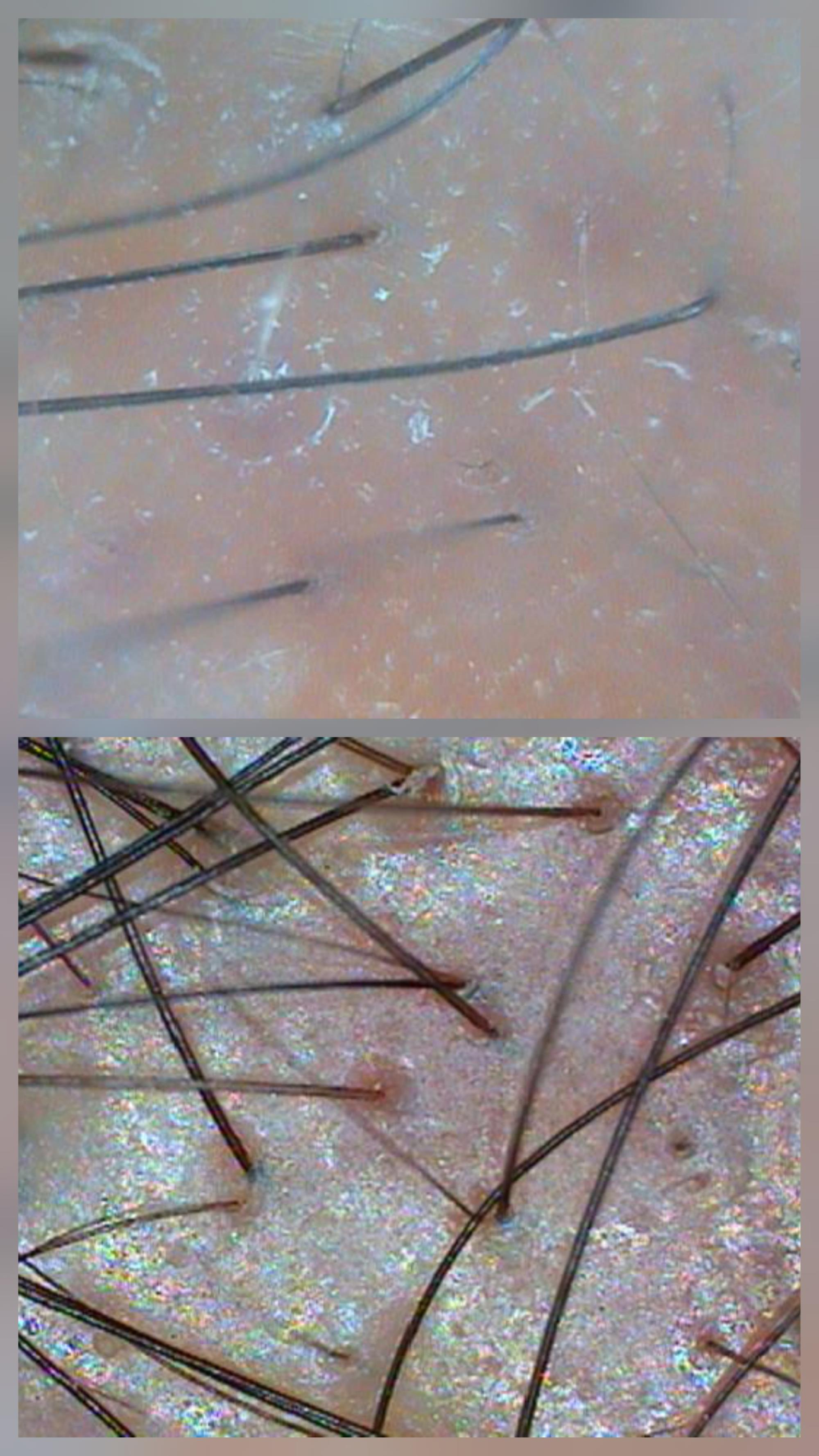

Supplement: Supplementary file 1 [file jcm-15-05055-s001.zip › Supplementary_File_S3_Trichoscopy_Images/S3_images/P2/loc1.png]

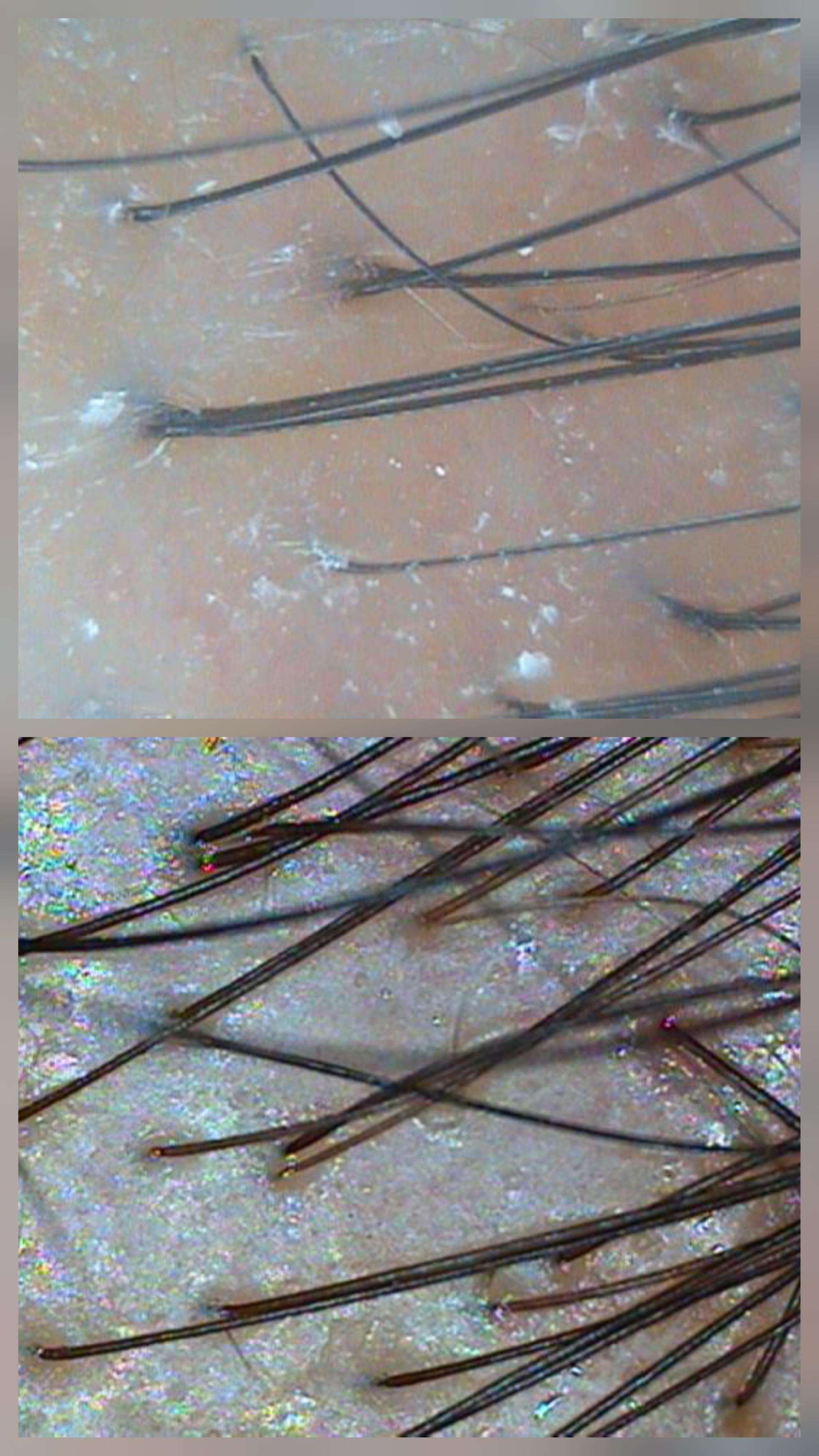

Supplement: Supplementary file 1 [file jcm-15-05055-s001.zip › Supplementary_File_S3_Trichoscopy_Images/S3_images/P2/loc2.png]

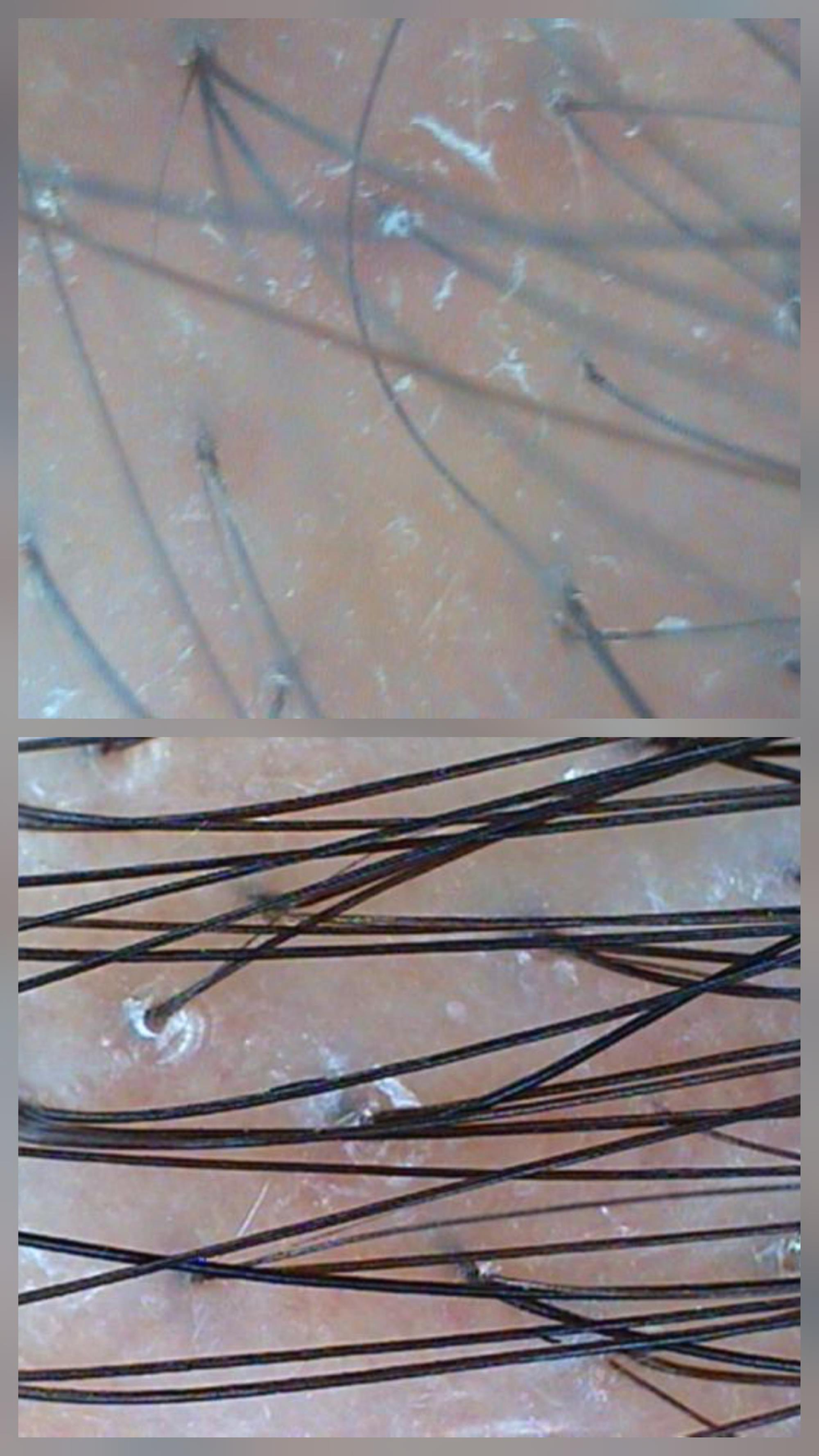

Supplement: Supplementary file 1 [file jcm-15-05055-s001.zip › Supplementary_File_S3_Trichoscopy_Images/S3_images/P2/loc3.png]

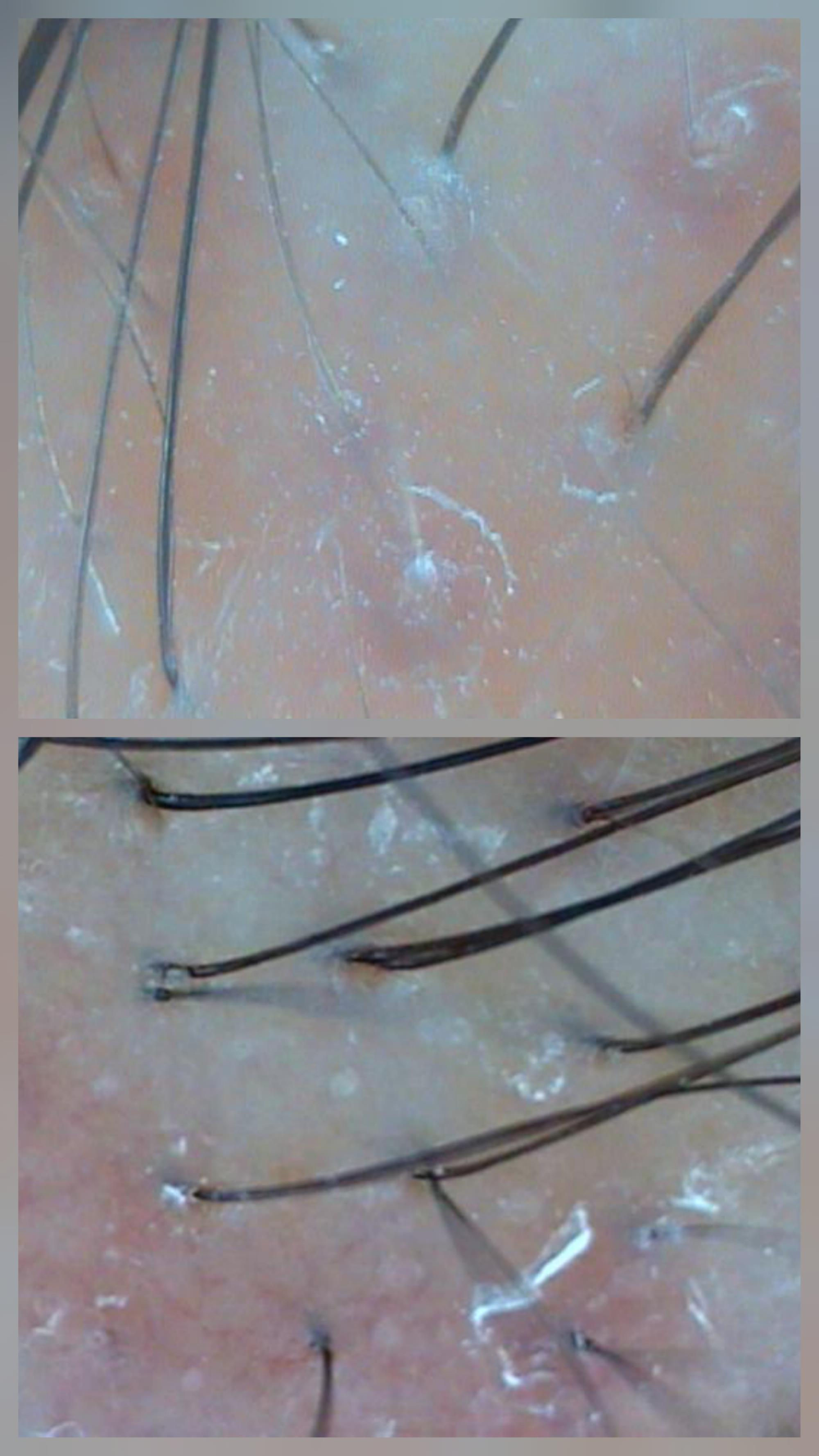

Supplement: Supplementary file 1 [file jcm-15-05055-s001.zip › Supplementary_File_S3_Trichoscopy_Images/S3_images/P2/loc4.png]

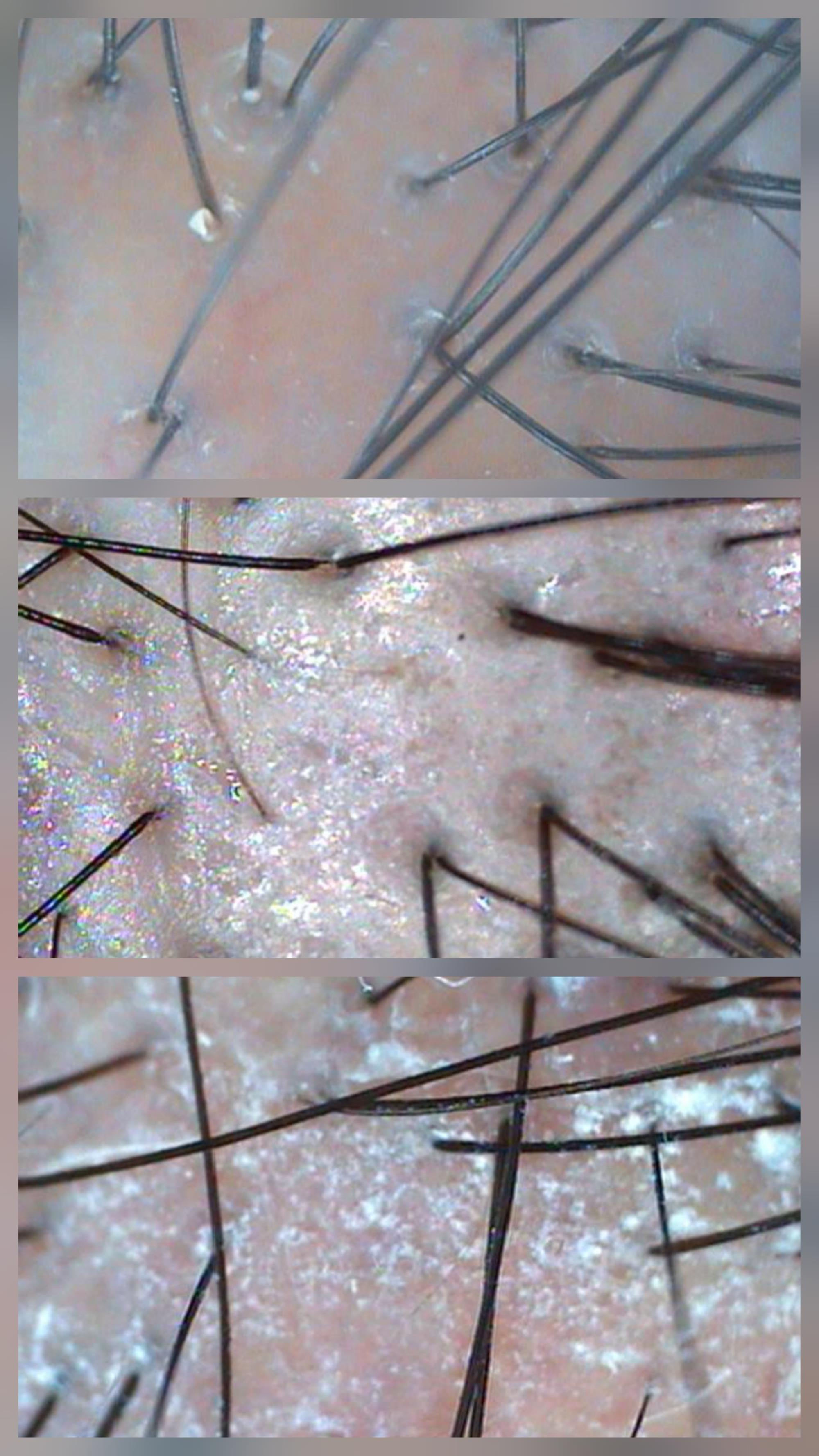

Supplement: Supplementary file 1 [file jcm-15-05055-s001.zip › Supplementary_File_S3_Trichoscopy_Images/S3_images/P3/loc1.png]

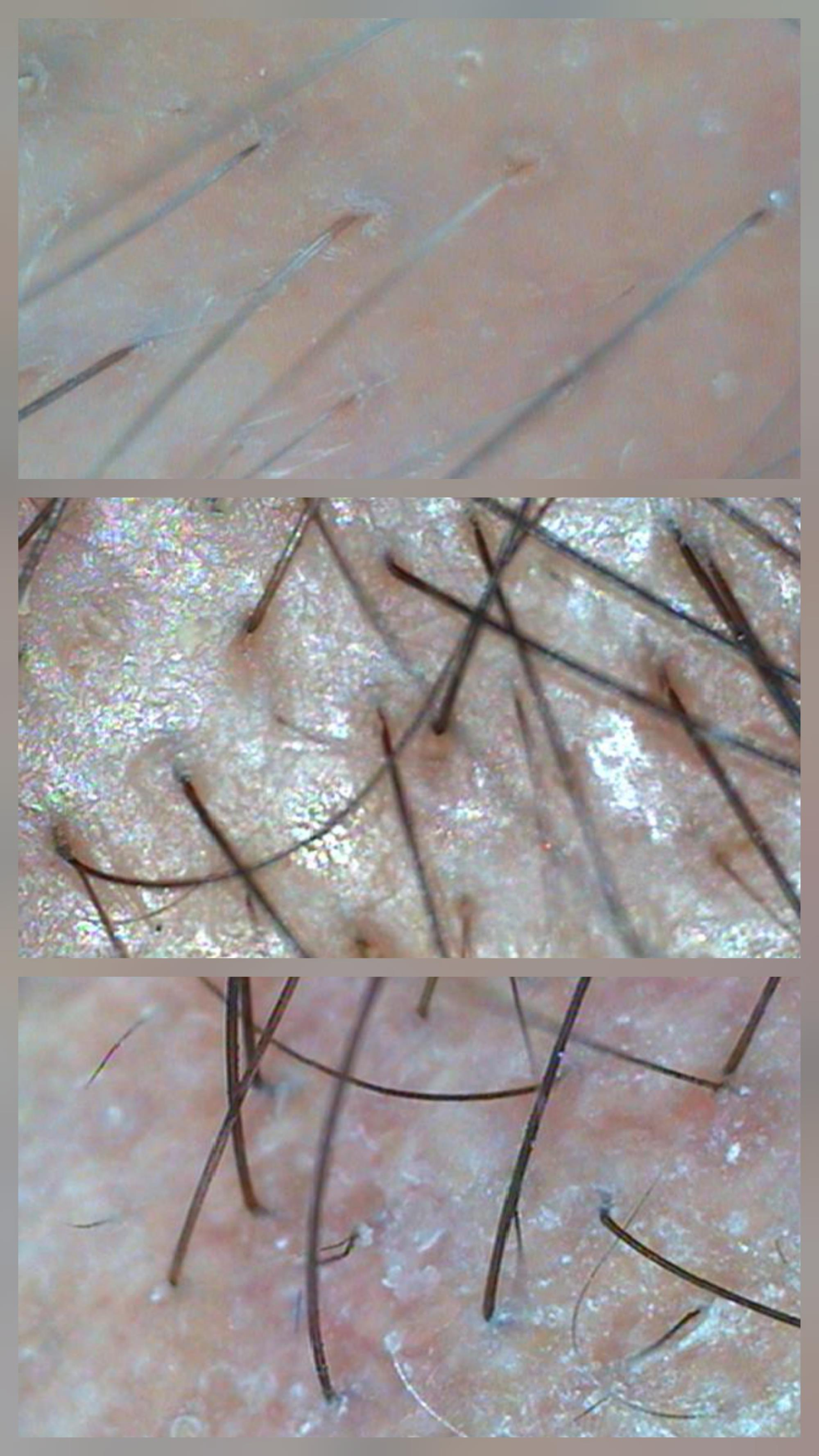

Supplement: Supplementary file 1 [file jcm-15-05055-s001.zip › Supplementary_File_S3_Trichoscopy_Images/S3_images/P3/loc2.png]

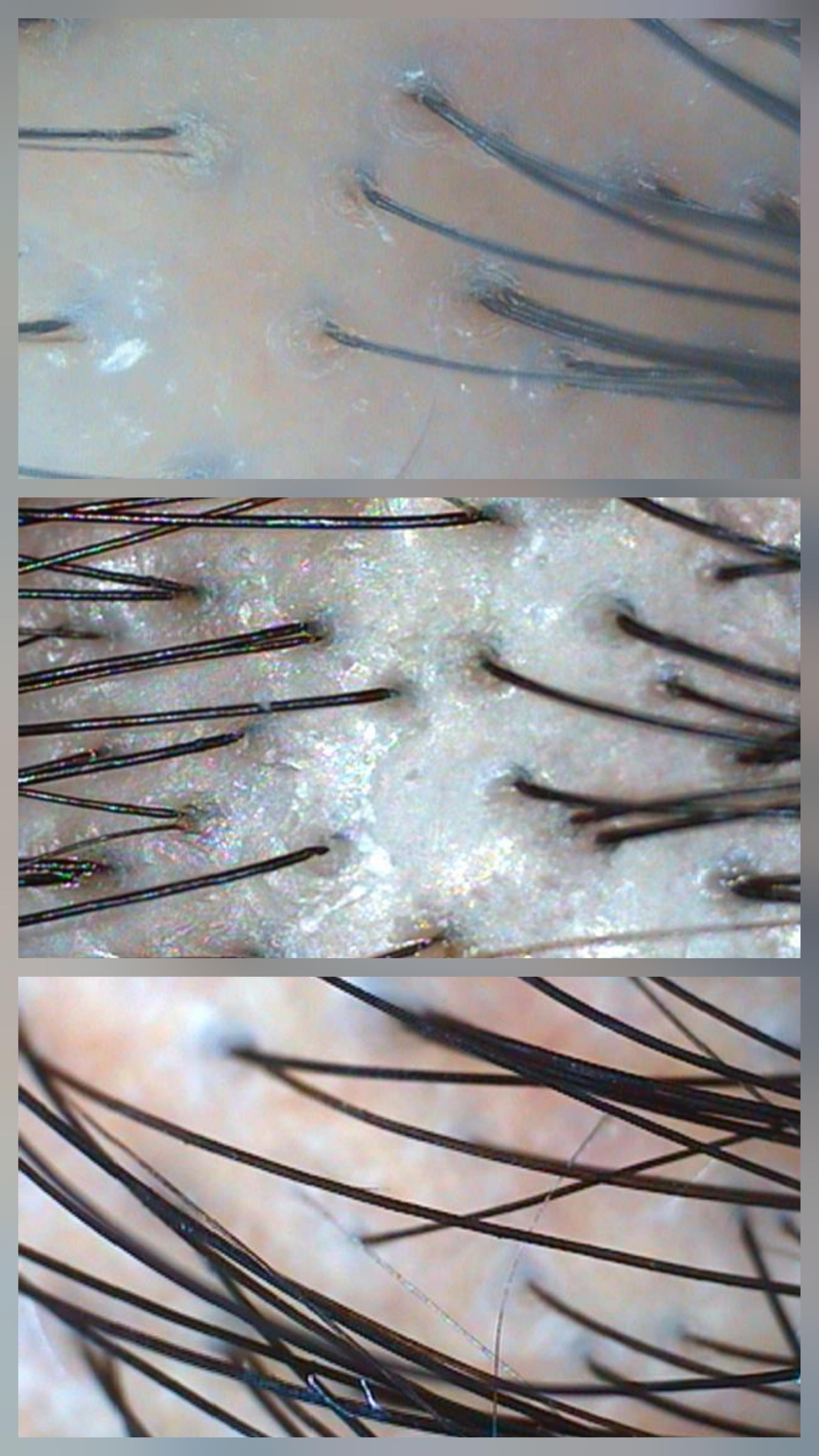

Supplement: Supplementary file 1 [file jcm-15-05055-s001.zip › Supplementary_File_S3_Trichoscopy_Images/S3_images/P3/loc3.png]

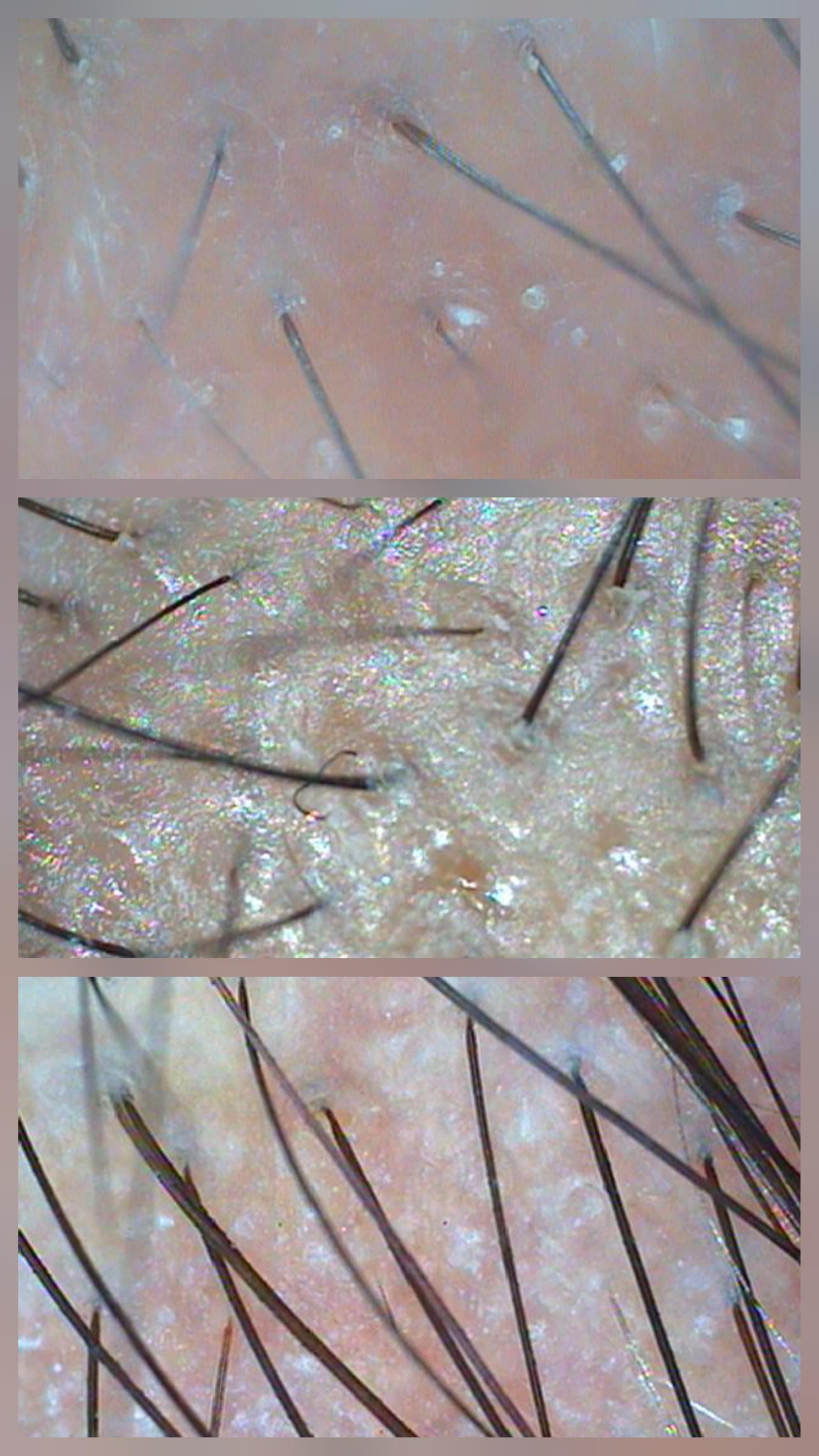

Supplement: Supplementary file 1 [file jcm-15-05055-s001.zip › Supplementary_File_S3_Trichoscopy_Images/S3_images/P3/loc4.png]

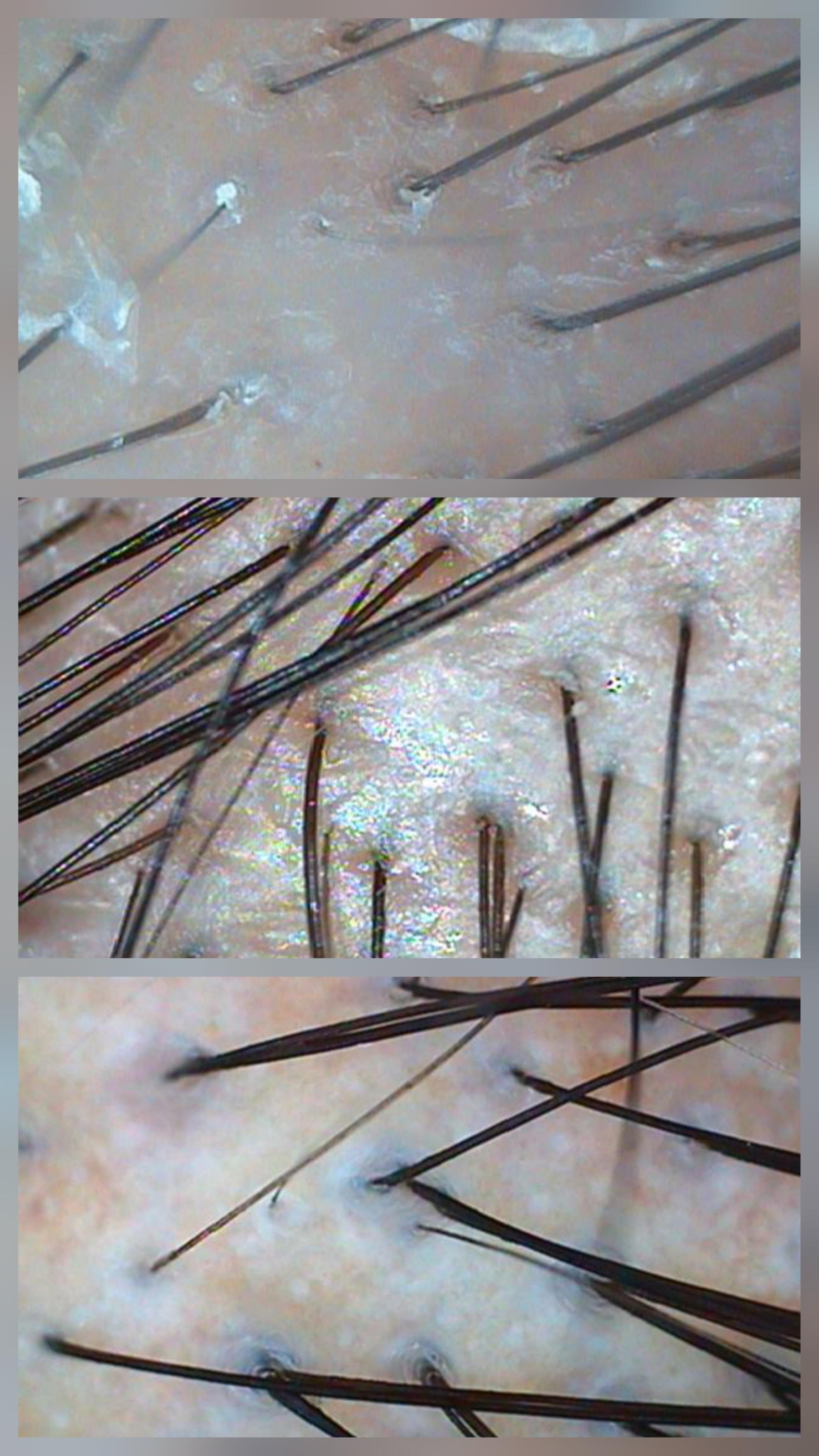

Supplement: Supplementary file 1 [file jcm-15-05055-s001.zip › Supplementary_File_S3_Trichoscopy_Images/S3_images/P3/loc5.png]

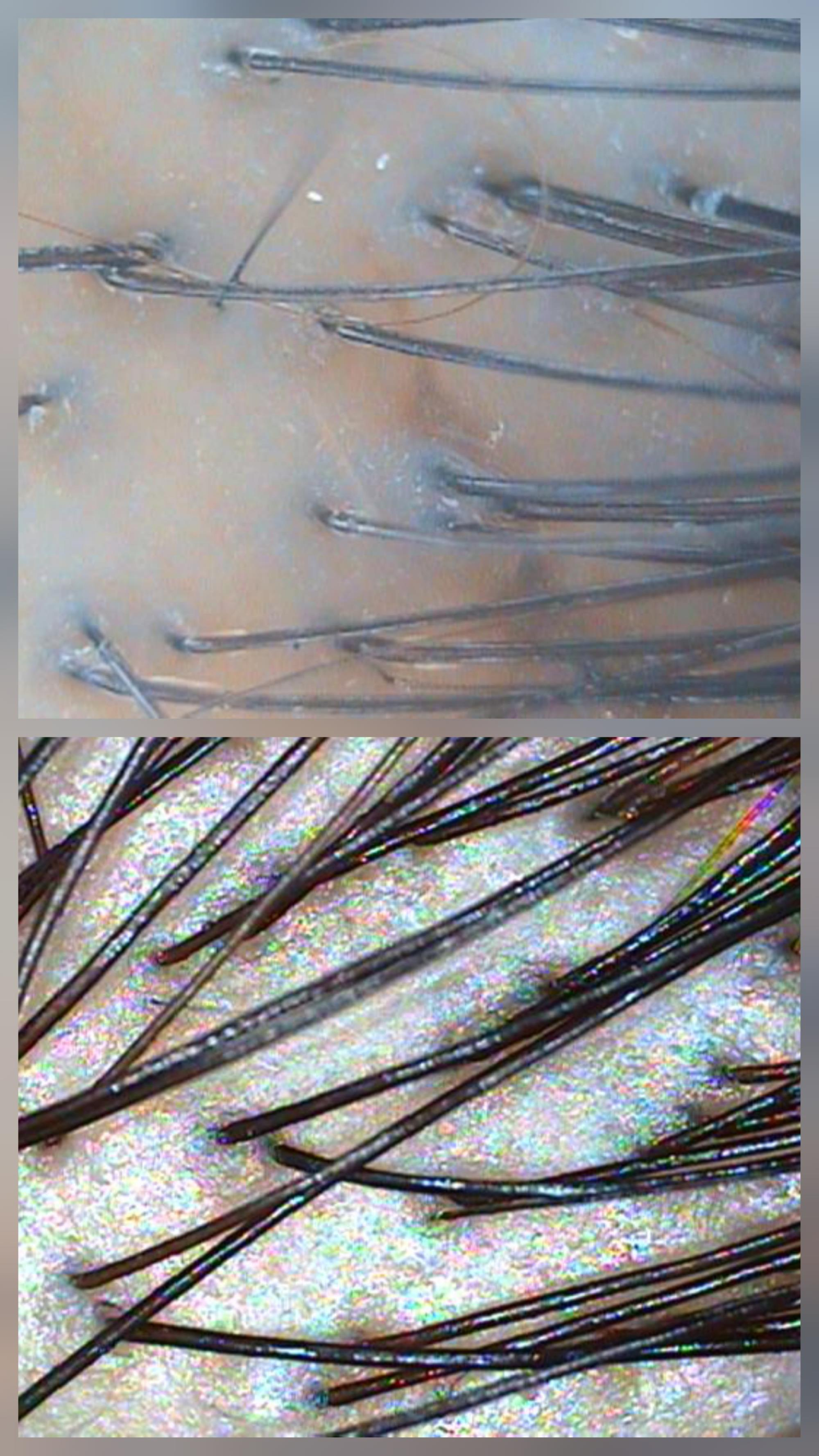

Supplement: Supplementary file 1 [file jcm-15-05055-s001.zip › Supplementary_File_S3_Trichoscopy_Images/S3_images/P4/loc1.png]

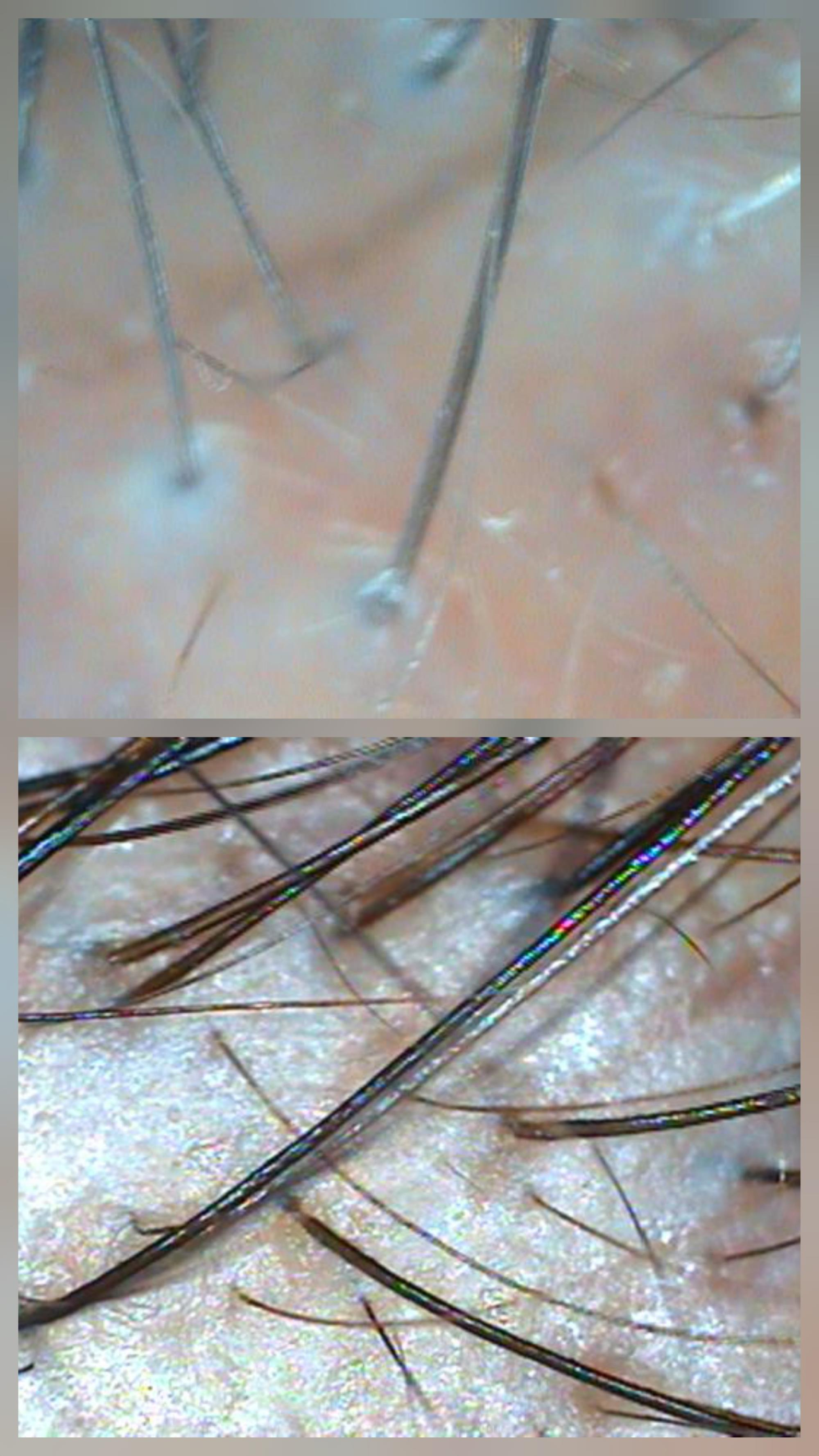

Supplement: Supplementary file 1 [file jcm-15-05055-s001.zip › Supplementary_File_S3_Trichoscopy_Images/S3_images/P4/loc2.png]

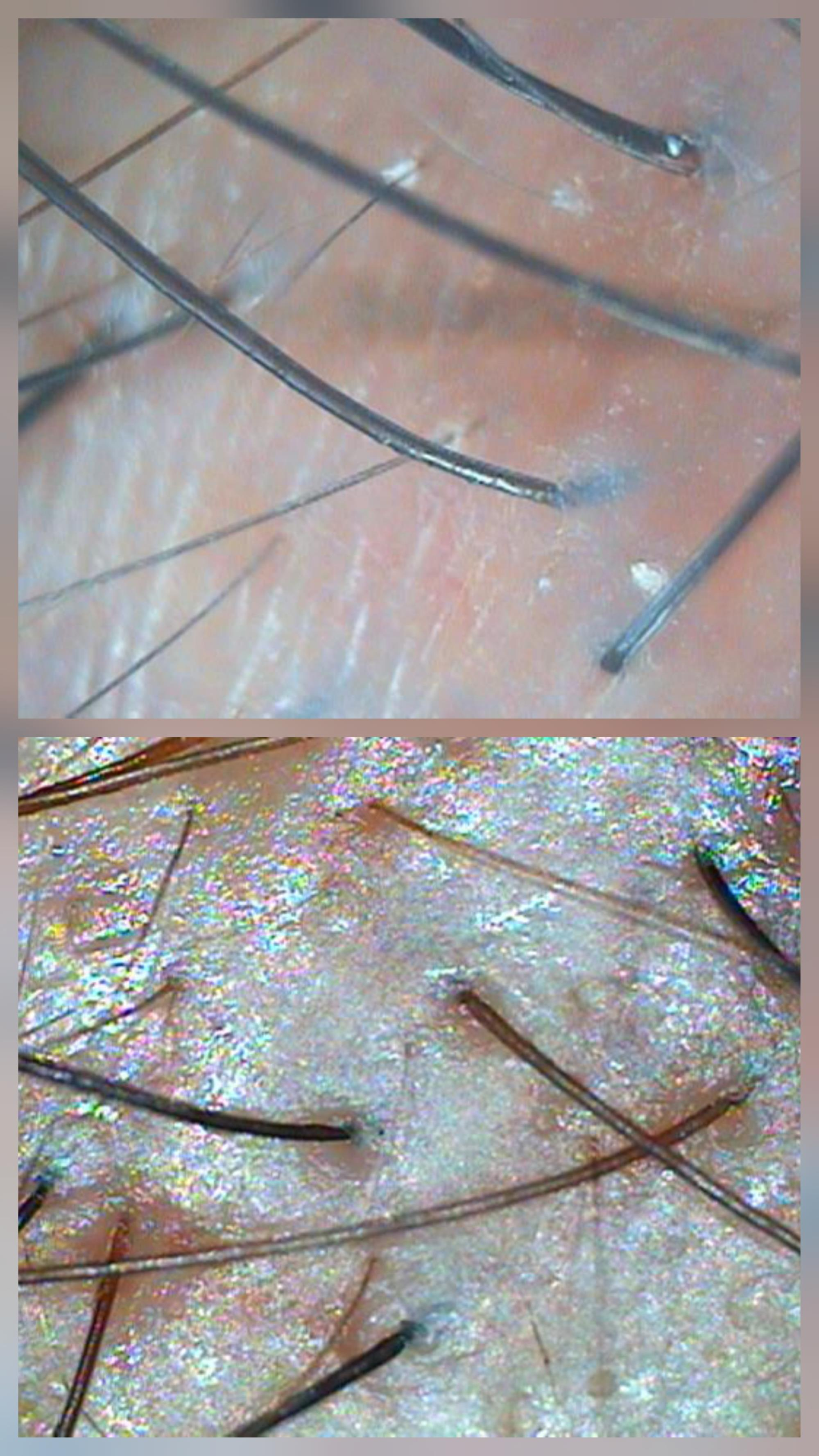

Supplement: Supplementary file 1 [file jcm-15-05055-s001.zip › Supplementary_File_S3_Trichoscopy_Images/S3_images/P4/loc3.png]

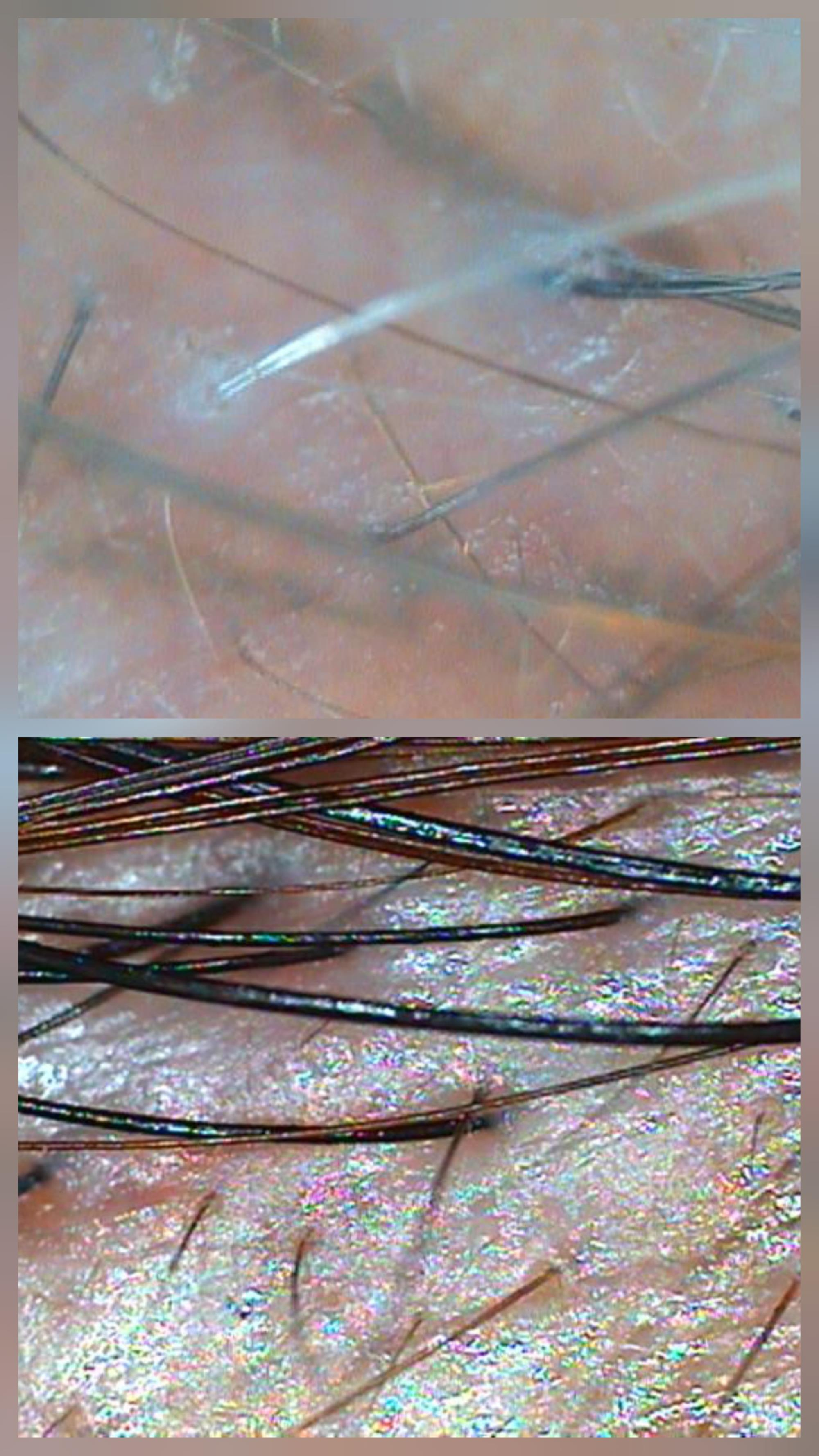

Supplement: Supplementary file 1 [file jcm-15-05055-s001.zip › Supplementary_File_S3_Trichoscopy_Images/S3_images/P4/loc4.png]

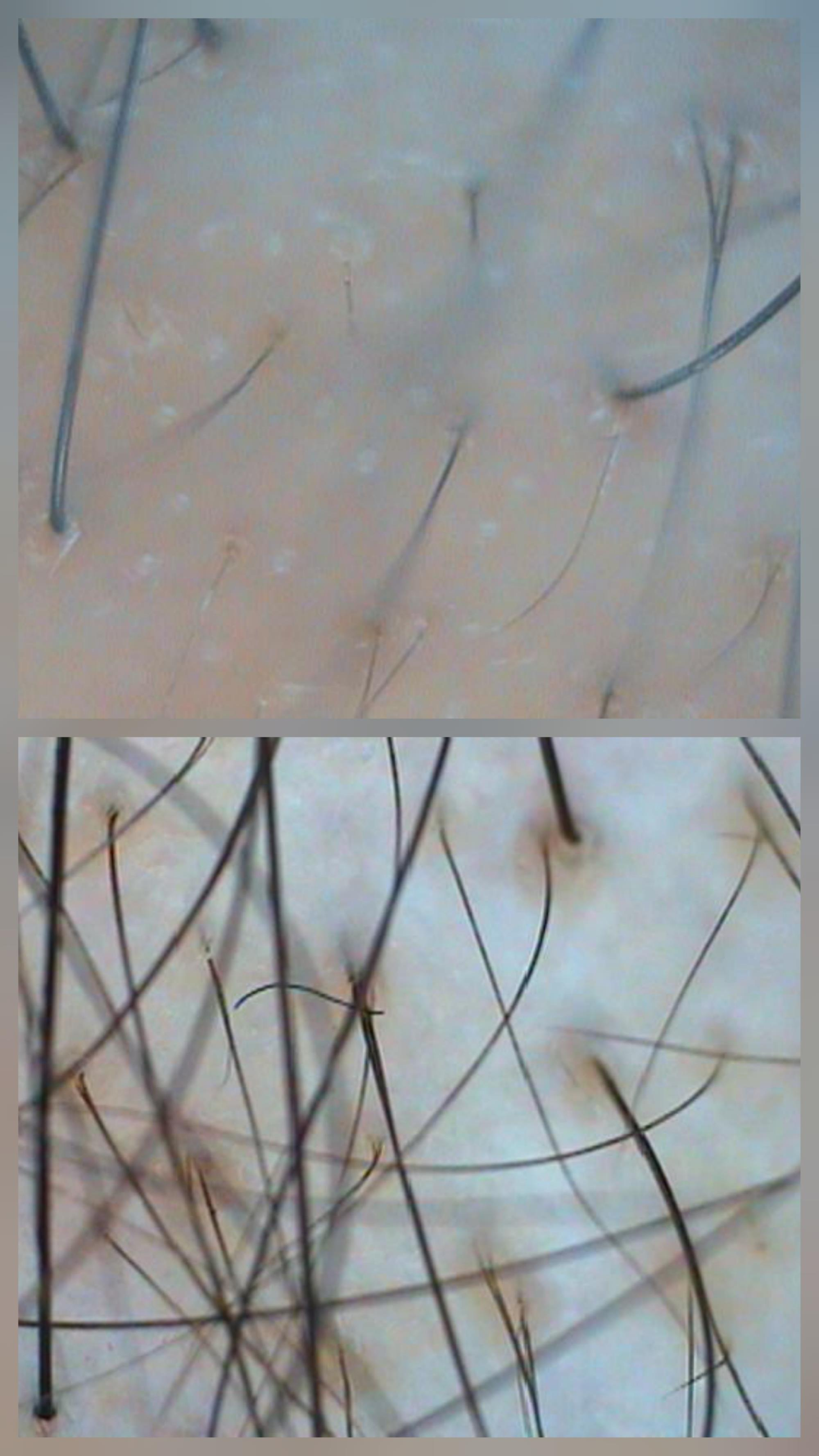

Supplement: Supplementary file 1 [file jcm-15-05055-s001.zip › Supplementary_File_S3_Trichoscopy_Images/S3_images/P4/loc5.png]

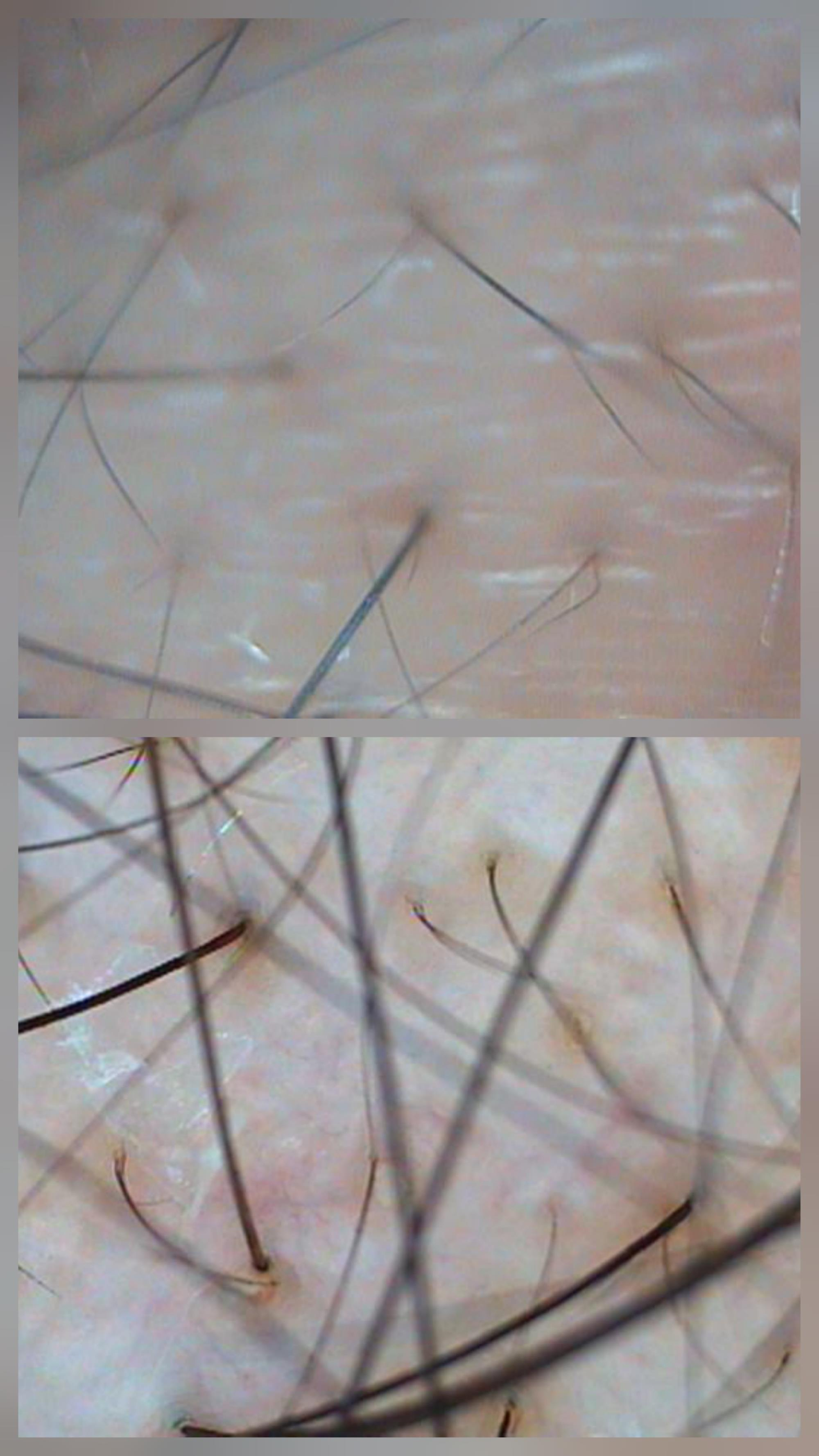

Supplement: Supplementary file 1 [file jcm-15-05055-s001.zip › Supplementary_File_S3_Trichoscopy_Images/S3_images/P5/loc1.png]

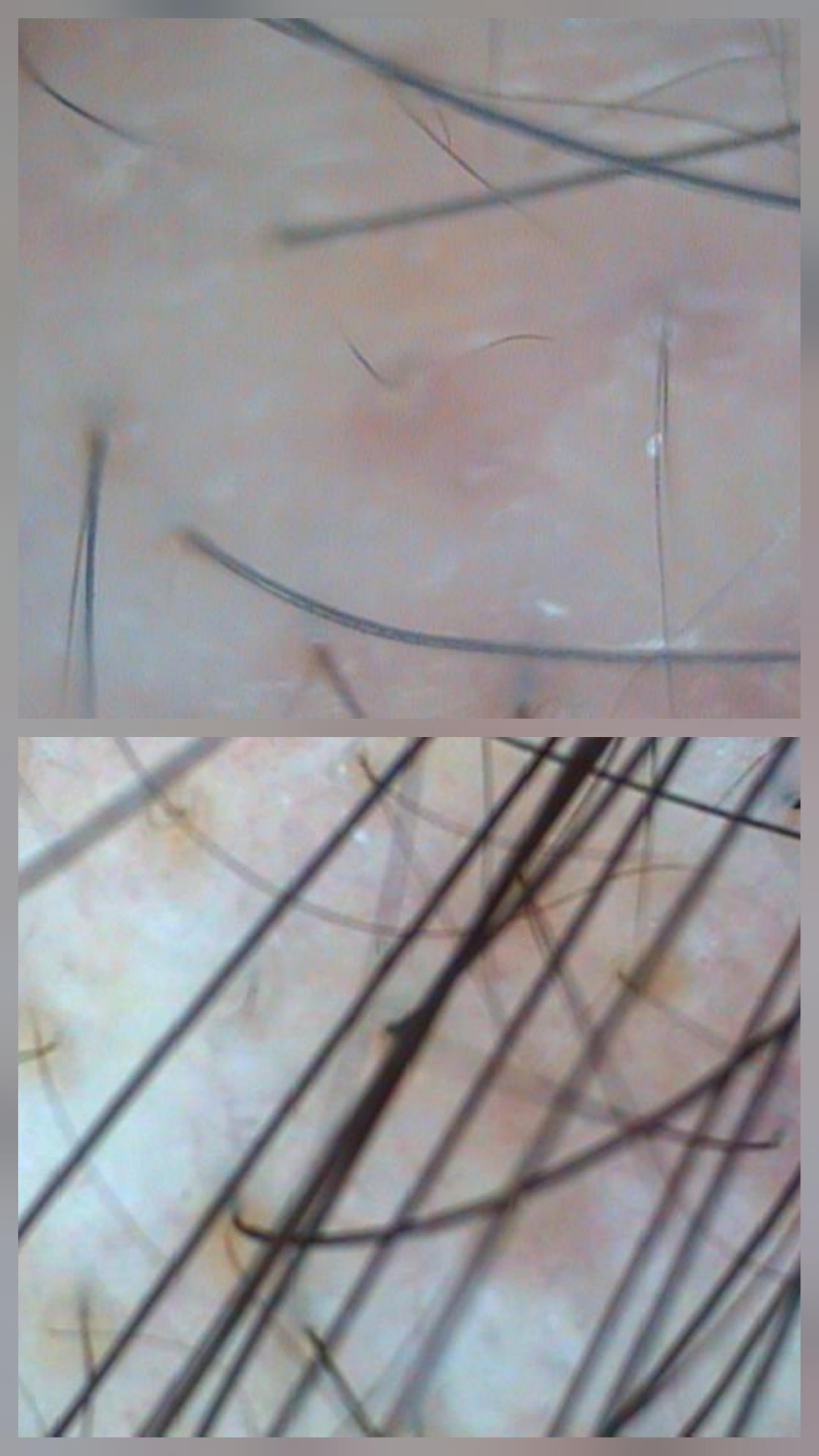

Supplement: Supplementary file 1 [file jcm-15-05055-s001.zip › Supplementary_File_S3_Trichoscopy_Images/S3_images/P5/loc2.png]

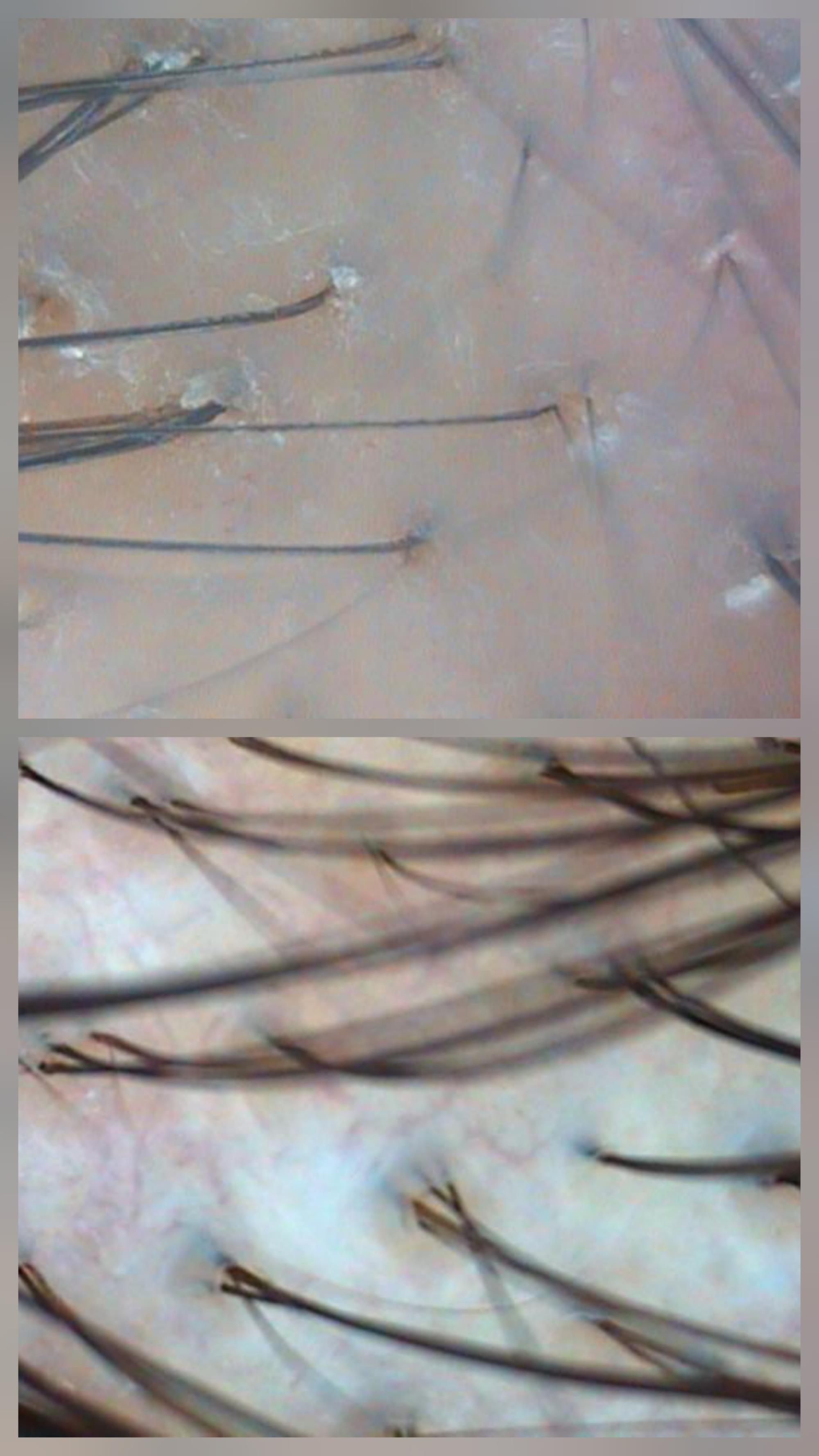

Supplement: Supplementary file 1 [file jcm-15-05055-s001.zip › Supplementary_File_S3_Trichoscopy_Images/S3_images/P5/loc3.png]

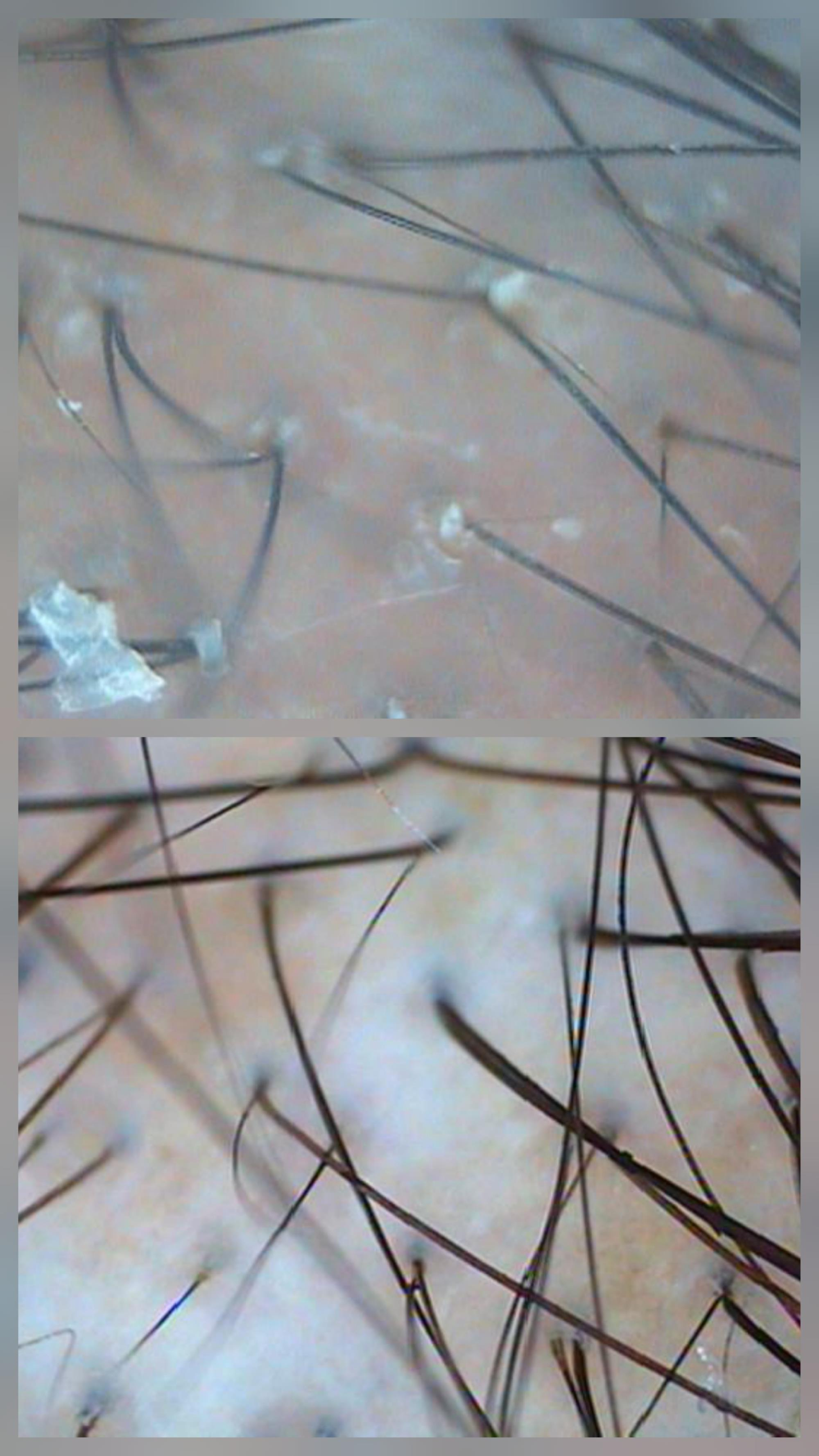

Supplement: Supplementary file 1 [file jcm-15-05055-s001.zip › Supplementary_File_S3_Trichoscopy_Images/S3_images/P5/loc5.png]

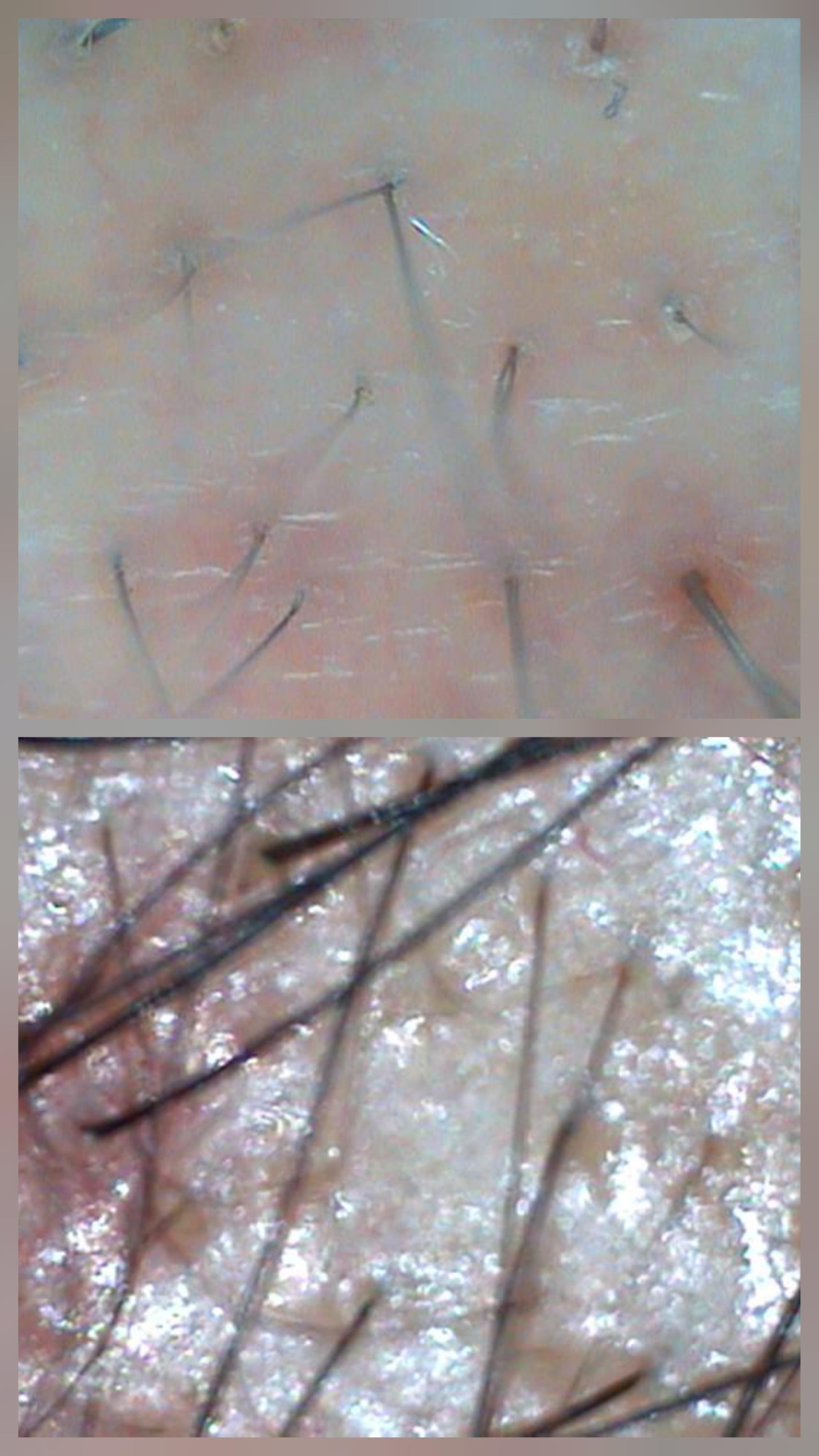

Supplement: Supplementary file 1 [file jcm-15-05055-s001.zip › Supplementary_File_S3_Trichoscopy_Images/S3_images/P6/loc1.png]

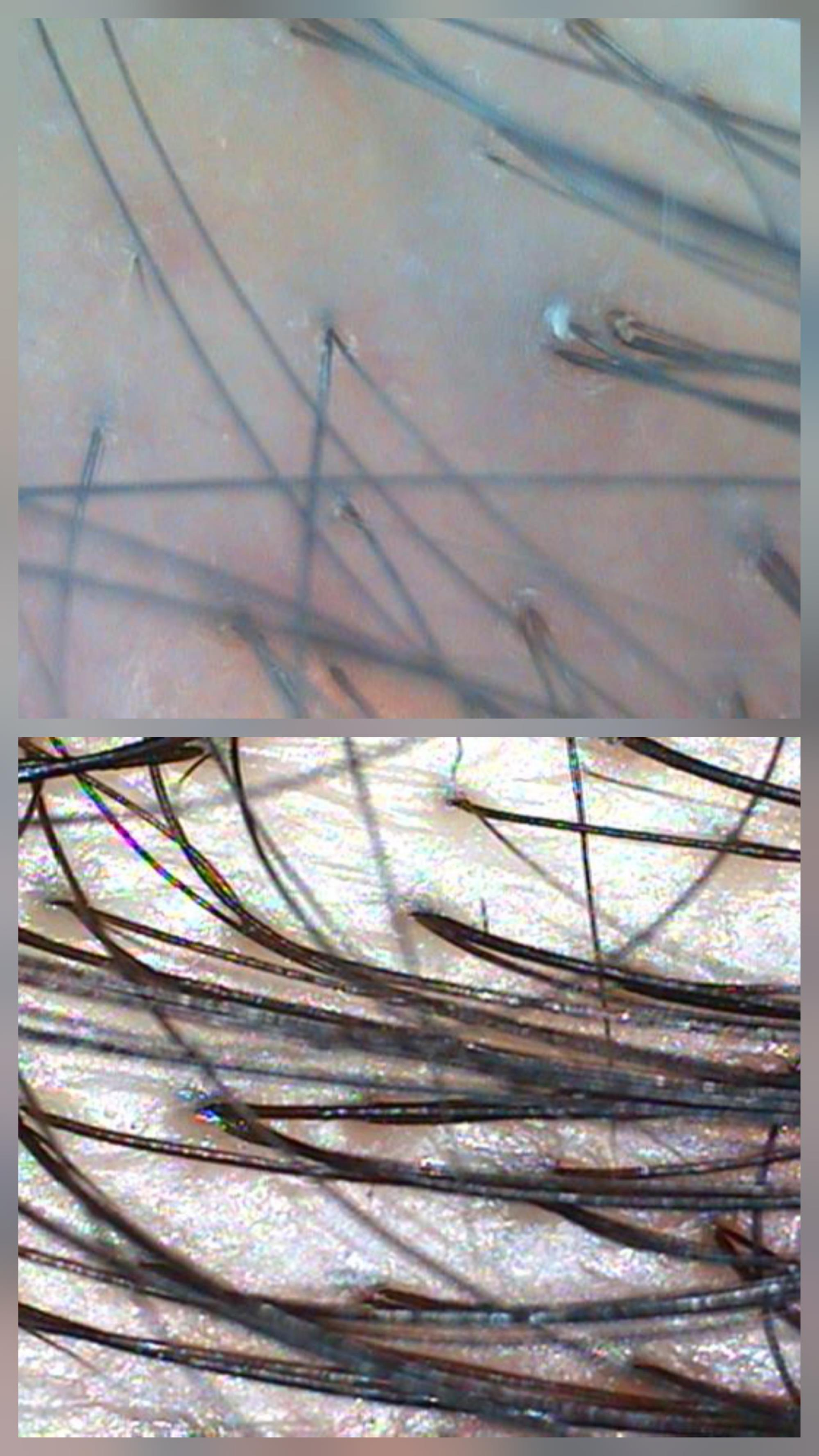

Supplement: Supplementary file 1 [file jcm-15-05055-s001.zip › Supplementary_File_S3_Trichoscopy_Images/S3_images/P6/loc2.png]

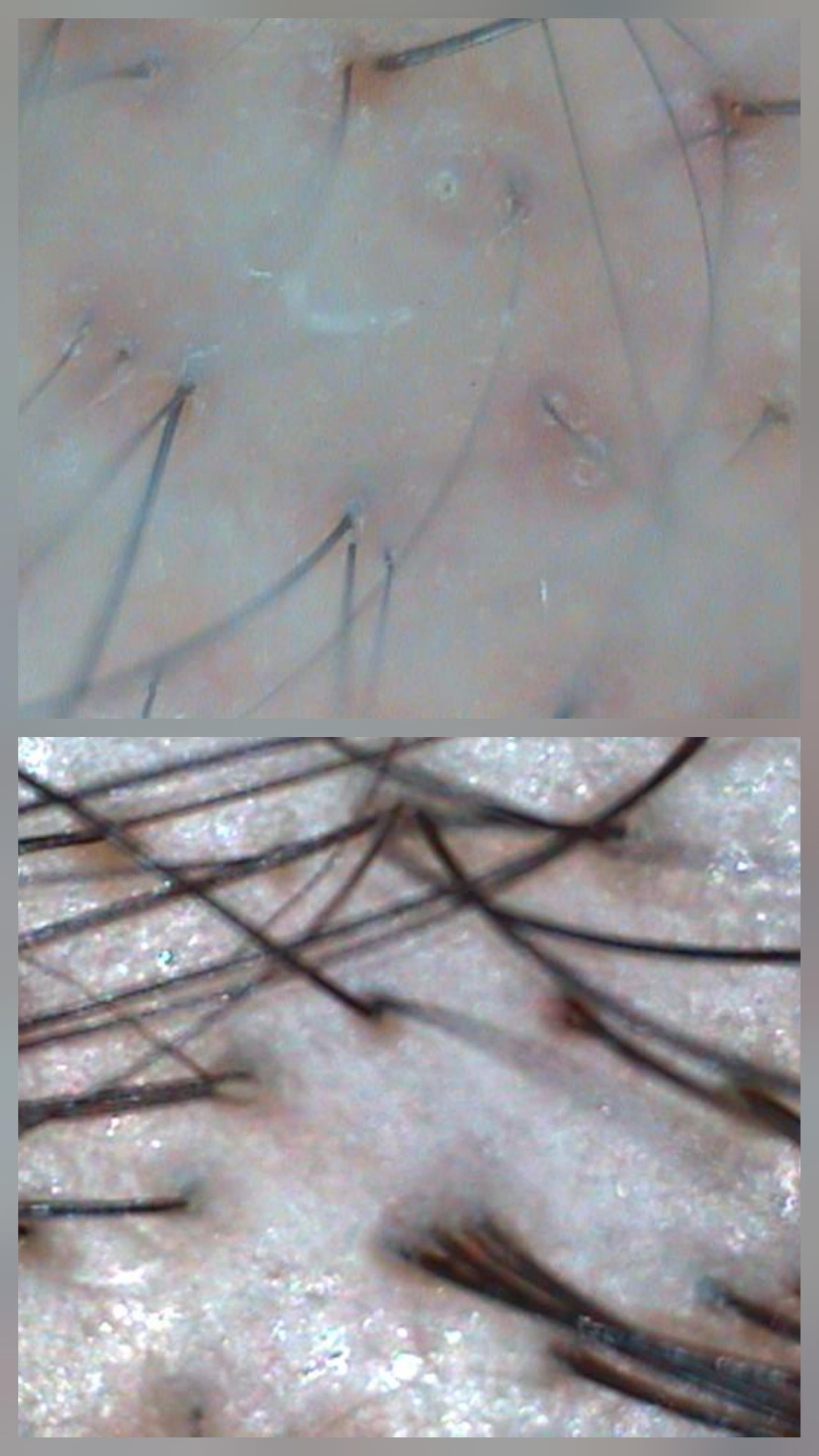

Supplement: Supplementary file 1 [file jcm-15-05055-s001.zip › Supplementary_File_S3_Trichoscopy_Images/S3_images/P6/loc3.png]

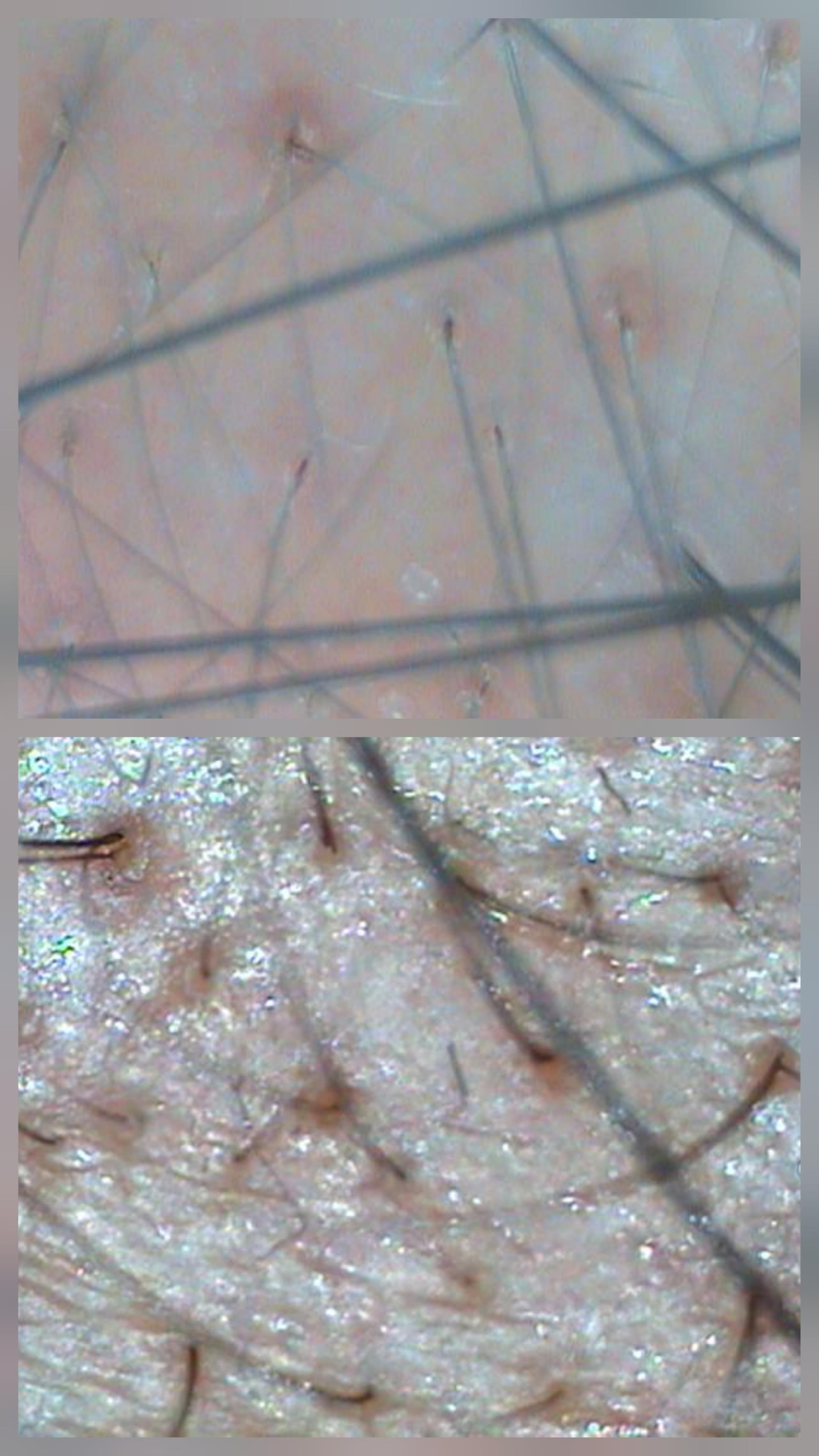

Supplement: Supplementary file 1 [file jcm-15-05055-s001.zip › Supplementary_File_S3_Trichoscopy_Images/S3_images/P6/loc4.png]

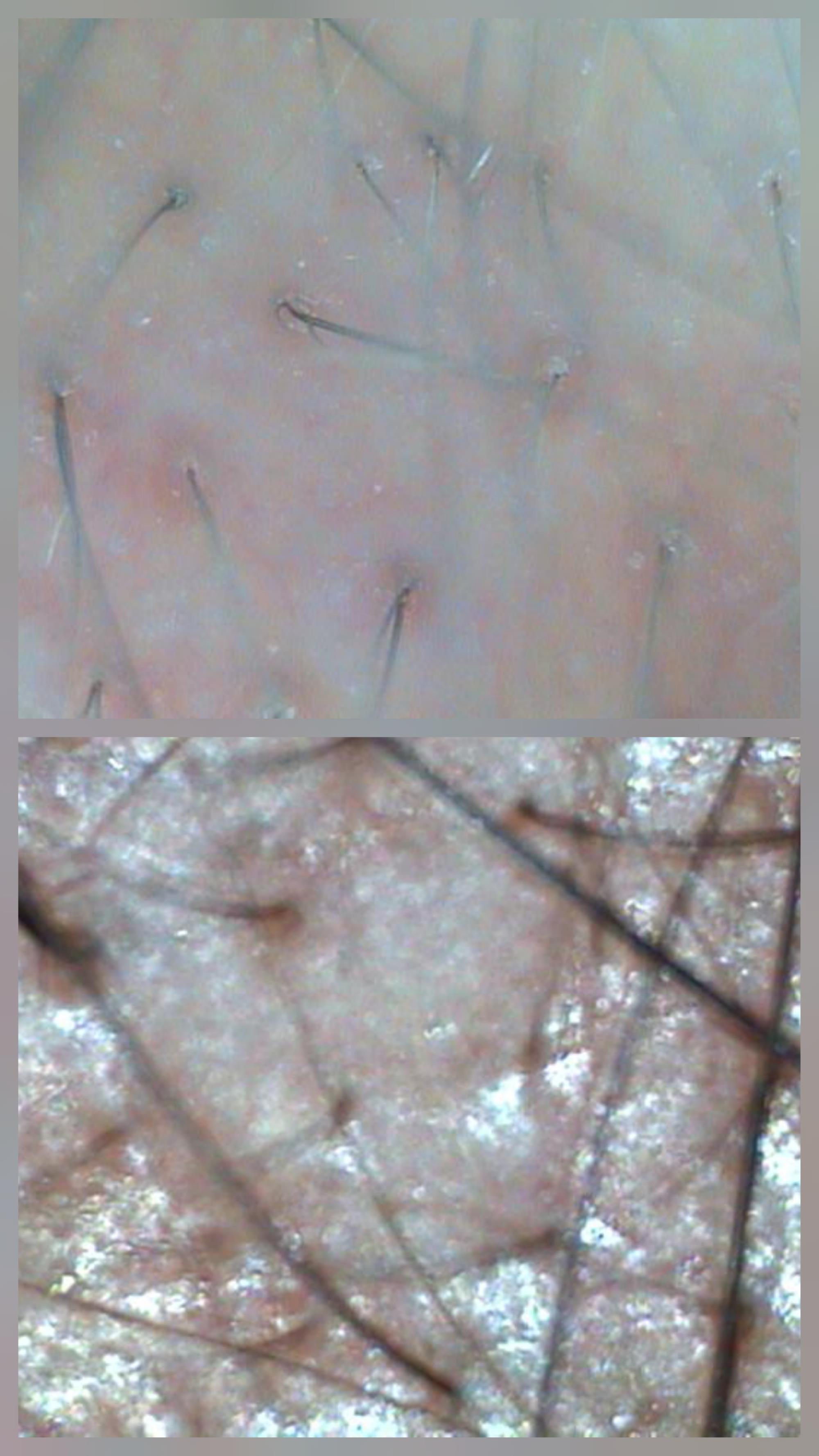

Supplement: Supplementary file 1 [file jcm-15-05055-s001.zip › Supplementary_File_S3_Trichoscopy_Images/S3_images/P6/loc5.png]

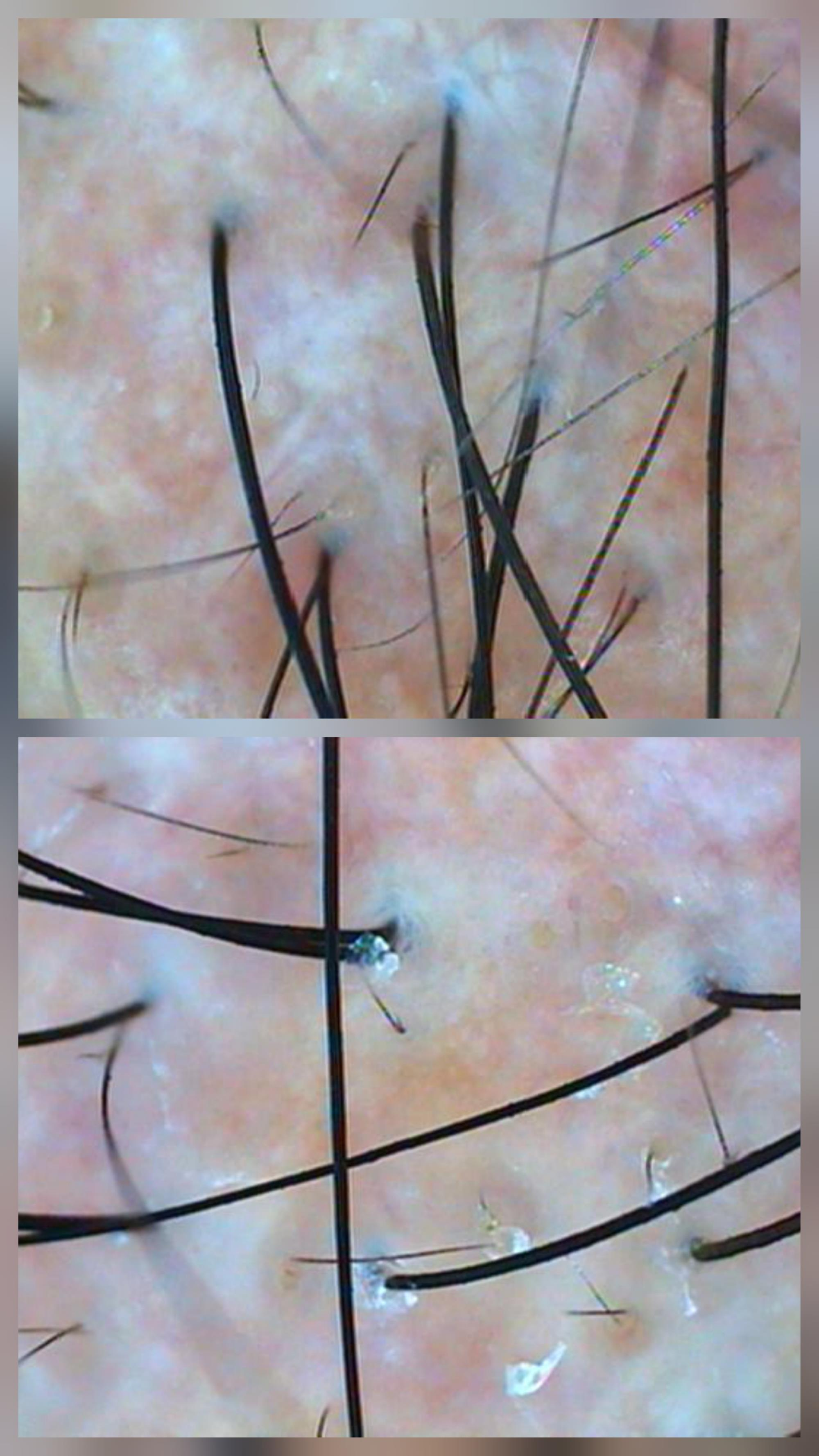

Supplement: Supplementary file 1 [file jcm-15-05055-s001.zip › Supplementary_File_S3_Trichoscopy_Images/S3_images/P7/loc1.png]

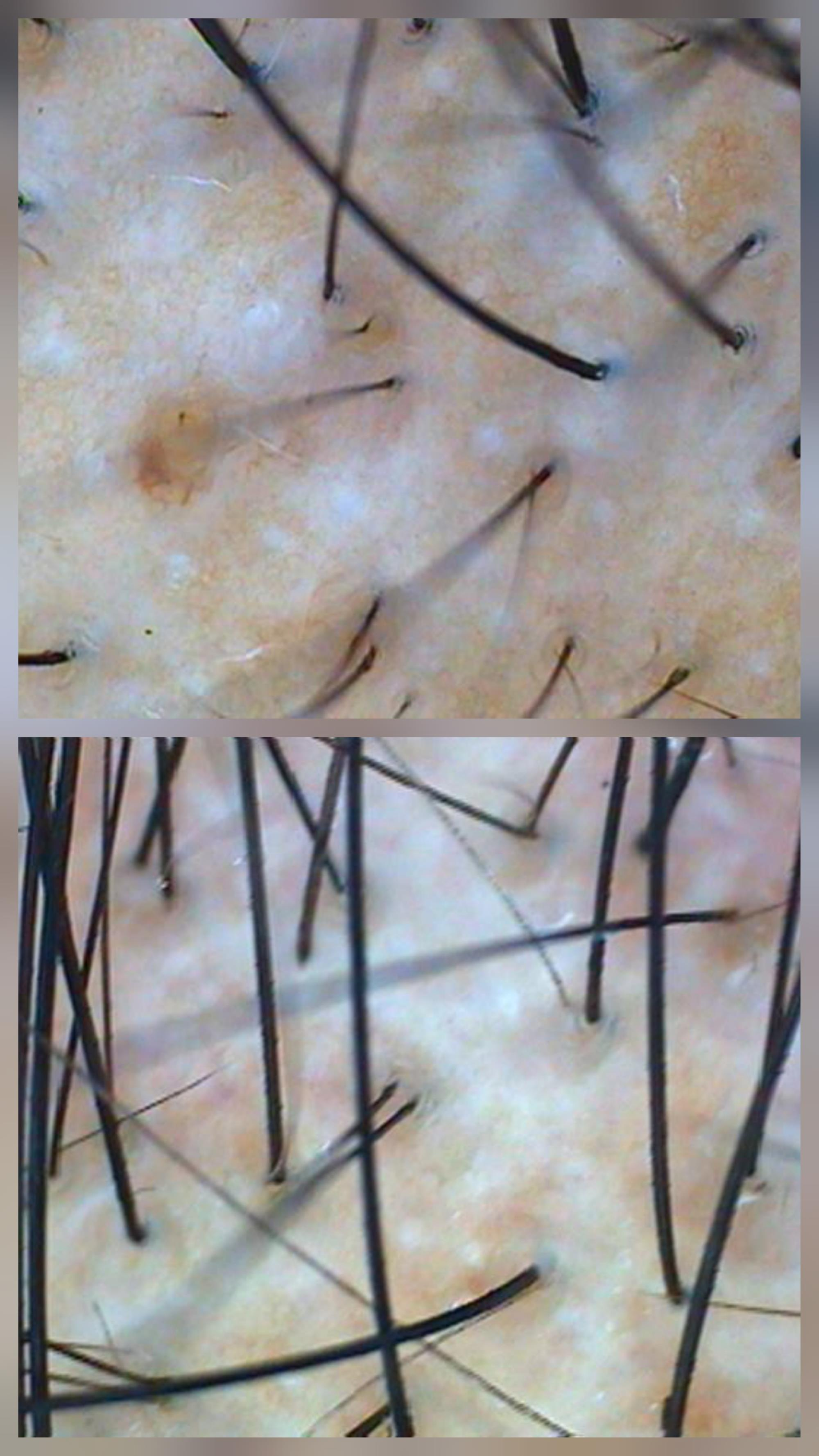

Supplement: Supplementary file 1 [file jcm-15-05055-s001.zip › Supplementary_File_S3_Trichoscopy_Images/S3_images/P7/loc2.png]

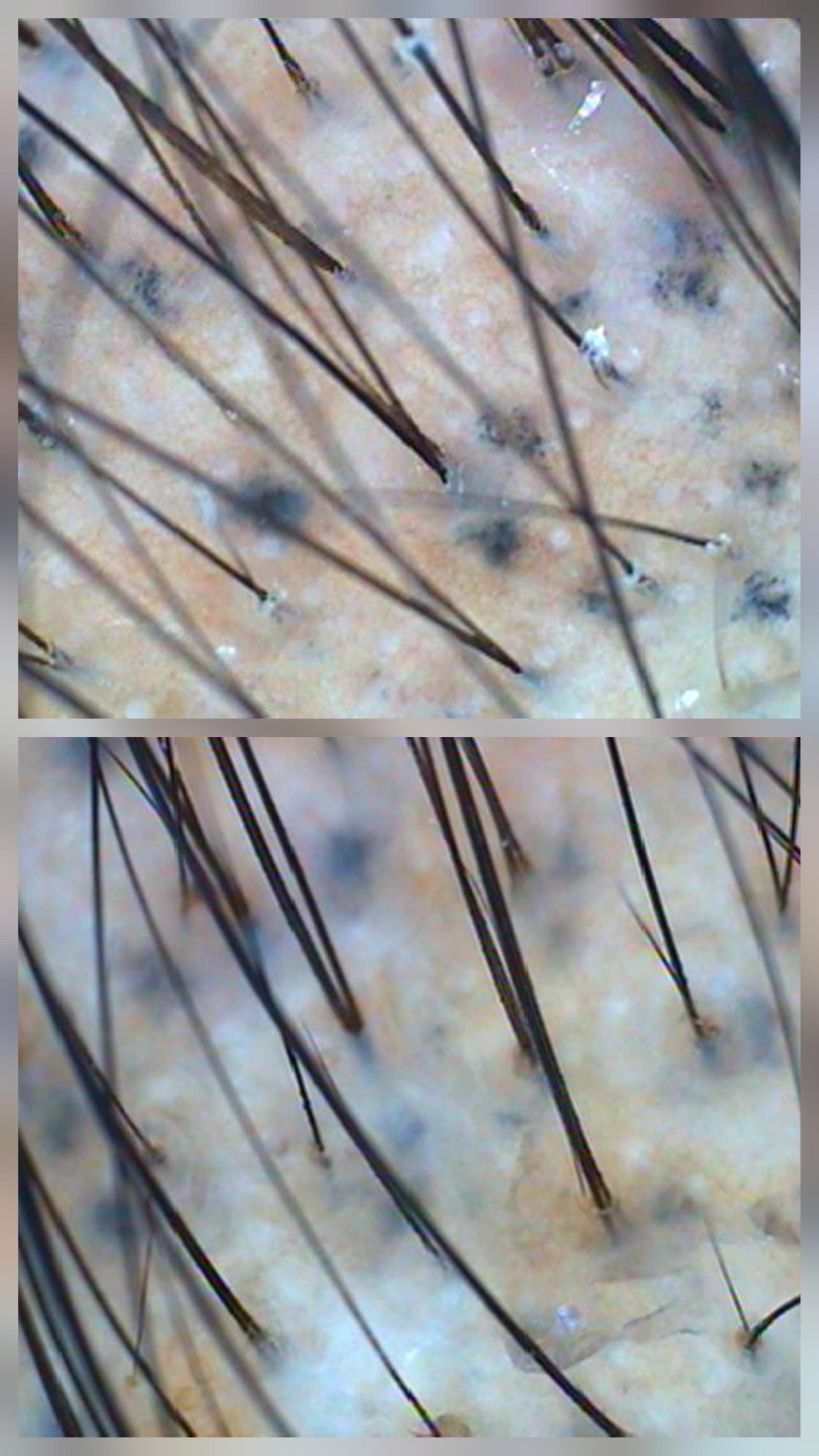

Supplement: Supplementary file 1 [file jcm-15-05055-s001.zip › Supplementary_File_S3_Trichoscopy_Images/S3_images/P7/loc3.png]

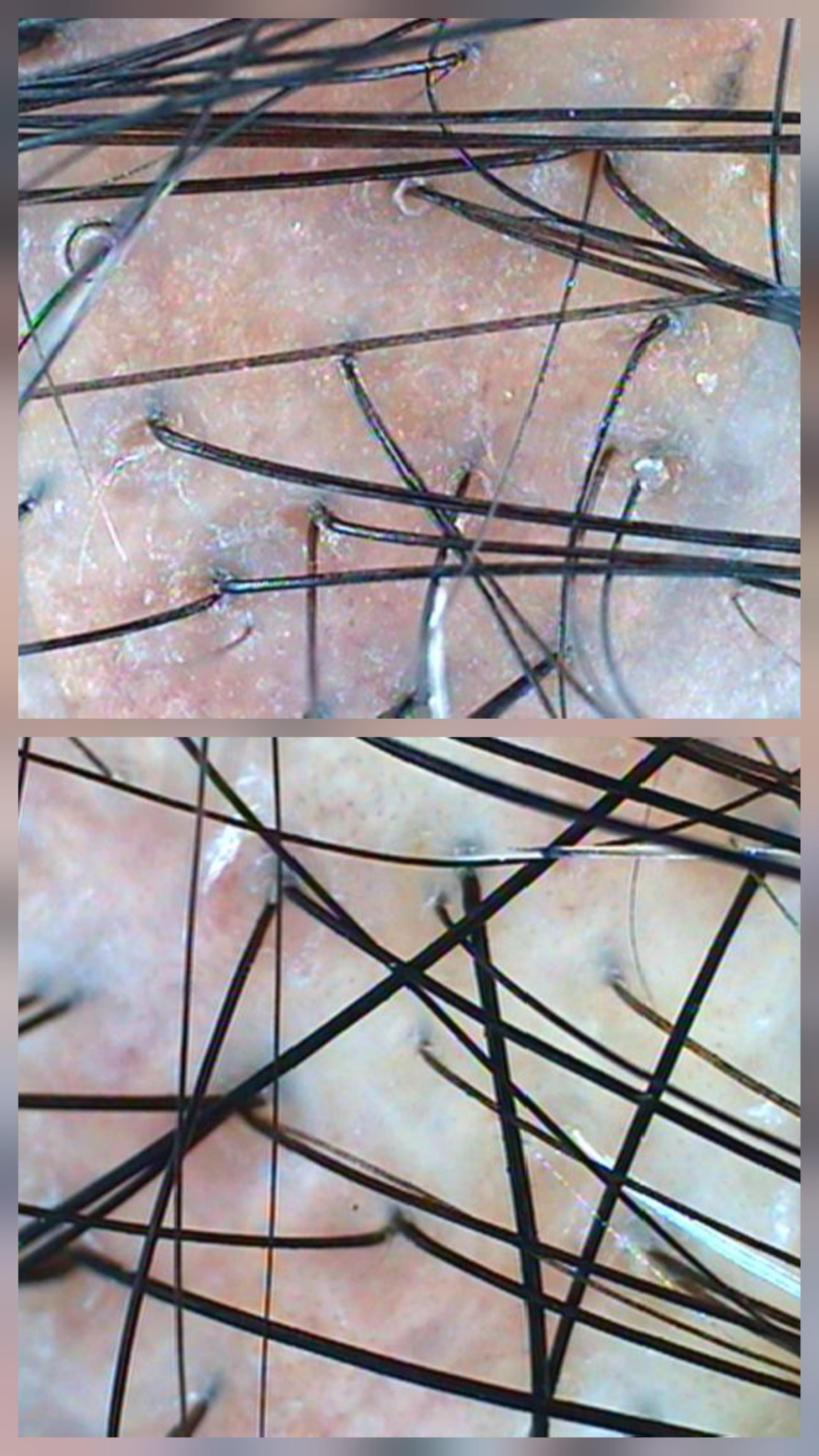

Supplement: Supplementary file 1 [file jcm-15-05055-s001.zip › Supplementary_File_S3_Trichoscopy_Images/S3_images/P7/loc4.png]

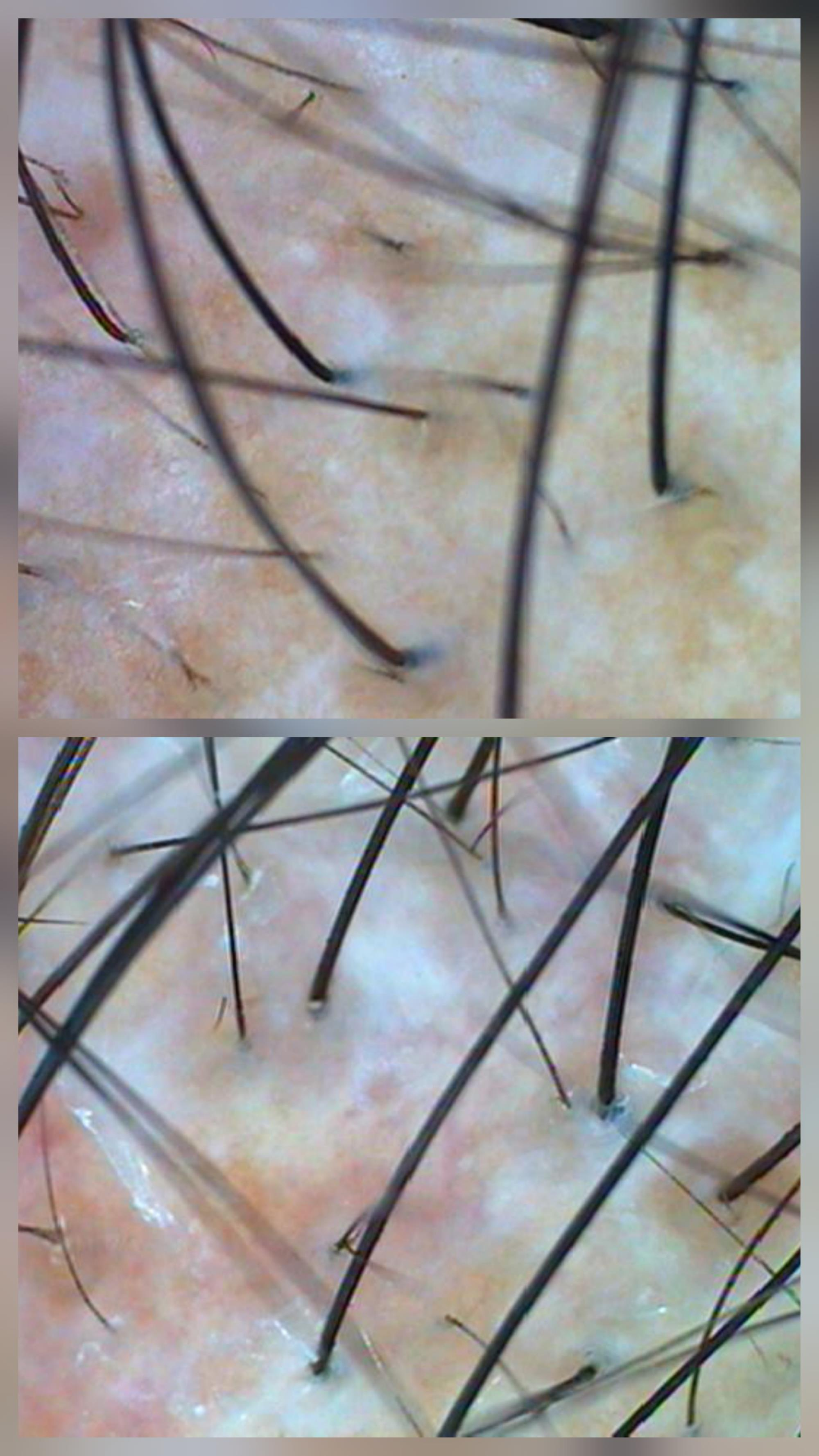

Supplement: Supplementary file 1 [file jcm-15-05055-s001.zip › Supplementary_File_S3_Trichoscopy_Images/S3_images/P7/loc5.png]
